# Highly Pathogenic Avian Influenza A (H5N1) Caused Mass Death Among Black‐Legged Kittiwakes (*Rissa tridactyla*) in Norway, 2023

**DOI:** 10.1155/tbed/2963364

**Published:** 2026-02-18

**Authors:** Grim Rømo, Caroline Piercey Åkesson, Tone Kristin Reiertsen, Johanna Hol Fosse, Cathrine Arnason Bøe, Lars Austbø, Johan Åkerstedt, Maryam Saghafian, Morten Helberg, Olav Hungnes, Britt Gjerset, Silje Granstad, Gørill Hogseth, Siri Løtvedt, Anne Døsen, Ragnhild Tønnessen

**Affiliations:** ^1^ Department of Animal Health, Welfare and Food Safety, Norwegian Veterinary Institute, Ås, Norway, vetinst.no; ^2^ Department of Analysis and Diagnostics, Norwegian Veterinary Institute, Ås, Norway, vetinst.no; ^3^ FRAM Centre, Norwegian Institute of Nature Research, Tromsø, Norway, nina.no; ^4^ BirdLife Norway, Trondheim, Norway; ^5^ Department of Virology, Norwegian Institute of Public Health, Oslo, Norway, fhi.no; ^6^ Northern Region, Norwegian Food Safety Authority, Vadsø, Norway; ^7^ Department of Regulations and Control, Norwegian Food Safety Authority, Oslo, Norway

**Keywords:** black-legged kittiwake, gulls, H5N1, highly pathogenic avian influenza, HPAI, Norway, One Health, pathology, receptors, virology

## Abstract

In 2023, highly pathogenic avian influenza (HPAI) heavily affected gulls in Europe. In July, a mass mortality event was reported in the black‐legged kittiwake (*Rissa tridactyla*) breeding colony at Ekkerøy in Northern Norway. The cause was confirmed to be infection with the HPAI H5N1 clade 2.3.4.4b virus, genotype EA‐2022‐BB. We describe the outbreak in kittiwakes, including pathological and virological investigations, and discuss the management and zoonotic potential. With more than 15,000 dead birds reported, we estimate that the outbreak caused a reduction in the kittiwake population at Ekkerøy of at least 50%. Diseased birds exhibited neurological signs. Necropsies of 10 birds revealed a peracute fatal systemic disease, with severe lesions in the brain and pancreas co‐localizing with viral RNA and antigen. Vascular expression of α2,3‐linked sialic acids (SAs) and viral RNA/antigen may reflect hematogenous viral spread. Further studies should investigate the long‐term impact of HPAI on kittiwake populations.

## 1. Introduction

From autumn 2020 to 2023, three major highly pathogenic avian influenza (HPAI) epizootics caused by H5Nx clade 2.3.4.4b viruses hit Europe, causing millions of deaths among wild and domestic birds [1]. During this period, H5N1 became the predominant avian influenza virus (AIV) subtype and spread with migratory birds across continents, causing a panzootic [2, 3]. Several genotypes of H5N1 circulated in birds in Europe [4], with the Eurasian BB genotype (H5N1‐A/Herring_gull/France/22P015977/2022‐like), also known as EA‐2022‐BB, becoming predominant from February to October 2023 [5]. This genotype contains three H13 virus‐derived gene segments, likely contributing to adaptation in gull species [6].

In Norway, the BB genotype was first detected in a dead herring gull (*Larus argentatus*) in April 2023. However, by summer, the black‐legged kittiwakes (*Rissa tridactyla*) (hereafter referred to as kittiwakes) became the most severely affected species.

Kittiwakes are long‐lived seabirds that breed in colonies on steep cliffs in the boreal and Arctic zones. They migrate along the Eastern Atlantic Ocean, with most birds wintering in the Western Atlantic. This species is listed as vulnerable on the IUCN Red List and as endangered on the Norwegian Red List [7]. The Ekkerøy colony (70°4′ N, 30°7′ E) in Vadsø municipality, Northern Norway, is the largest on mainland Norway, with an estimated 17,000 breeding pairs in 2012 [8].

Despite previous detections of AIVs and antibodies against non‐H5 AIV subtypes in kittiwakes [9–12], limited knowledge exists about the occurrence of HPAI H5 viruses in this species [13], and no studies have described the pathology or avian influenza receptor distribution. The impact of HPAI virus (HPAIV) infection on kittiwake colonies has, up until now, been largely unknown.

Recent spillover of HPAI H5N1 clade 2.3.4.4b viruses to wild and domestic mammals [1], such as farmed fur animals [14, 15], marine mammals [16], ruminants, and cats [17], raises concern for zoonotic transmission. Although human cases remain sporadic [18], increased circulation among domestic mammals elevates the pandemic potential, underscoring the importance of a One Health approach, including as part of an outbreak management plan.

In July 2023, HPAI caused a mass mortality event in the Ekkerøy kittiwake colony. We describe clinical signs, pathology, and virus receptor distribution in affected birds. Further, we characterize the causative H5N1 virus, including screening for mammalian‐adaptive mutations, estimate mortality in the colony, and discuss mitigation efforts and recommendations for future outbreak management.

## 2. Materials and Methods

Section 2 gives a brief overview of the material and methods used, with further details provided in Supporting Information 1: Appendix 1.

Confirmed HPAIV detections in kittiwakes in Norway from May 1 to July 31, 2023, were extracted from surveillance data from the Norwegian Veterinary Institute (NVI). Kittiwake mortality data were obtained from the County Governor of Troms and Finnmark. To estimate the number of kittiwake pairs breeding at Ekkerøy prior to the outbreak, an ad hoc survey of occupied nests was conducted by the Norwegian Institute of Nature Research and the Norwegian Nature Inspectorate on July 27, 2023.

Clinical observations of kittiwakes were mainly conducted at the breeding colony at Ekkerøy and at the river outlet of Storelva, 3.2 km north of the colony, where a high number of birds gathered. To ensure optimal sample quality, necropsy and sample collection were performed from 10 recently deceased kittiwakes. The birds were inspected, measured, and photographed, and their body condition score was assessed. From each bird, tracheal and cloacal swabs and a panel of tissues were collected for virological and pathological analyses, including histopathology, immunohistochemistry (IHC), RNAscope in situ hybridization (RNA‐ISH), *Maackia amurensis* lectin II (MAL‐II) staining, real‐time reverse transcription polymerase chain reaction (rRT‐PCR), virus whole genome sequencing (WGS), and sequence analyses.

## 3. Results

### 3.1. Extent of the Outbreak

**Figure 1:**
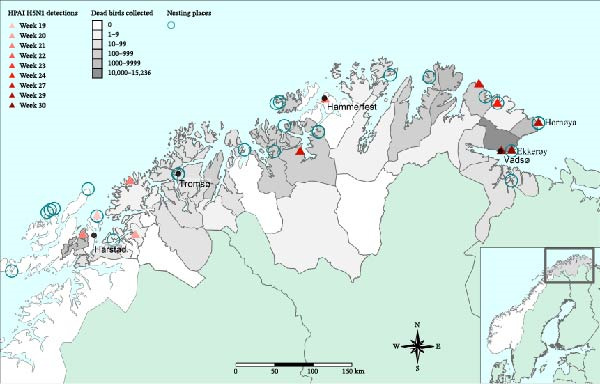
Weekly distribution of HPAIV H5N1 detections and reported mortality in black‐legged kittiwakes (*Rissa tridactyla*), from May to September 2023, Troms and Finnmark, Norway.

The first PCR detection of the HPAI H5N1 clade 2.3.4.4b virus in kittiwakes in Norway was recorded in May 2023, in Harstad (67°0′ N, 16°28′ E), where a smaller outbreak occurred. From May 1 to July 31, 2023, 32 of 40 dead kittiwakes submitted for analysis at the NVI tested positive for HPAI H5N1, confirming that HPAI was present in most breeding colonies in Troms and Finnmark (Figure 1).

Between July 28 and August 28, 2023, 24,594 wild bird carcasses, mainly kittiwakes, were recorded in Troms and Finnmark County in Northern Norway, representing a major HPAI outbreak (Supporting Information 1: Appendix 1 Table S1). Of these, 15,235 carcasses were collected in Vadsø municipality, where the kittiwake colony at Ekkerøy is located. Large numbers of additional dead kittiwakes were observed in areas near the colony at Ekkerøy, including coastal waters, roads, and rooftops, indicating that the true number of dead birds was even higher. For comparison, the total number of kittiwake breeding pairs at Ekkerøy in 2023 was estimated to be 7817 (15,634 kittiwakes) based on colony counts (Supporting Information 1: Appendix 1 Table S2).

### 3.2. Clinical Characteristics

**Figure 2:**
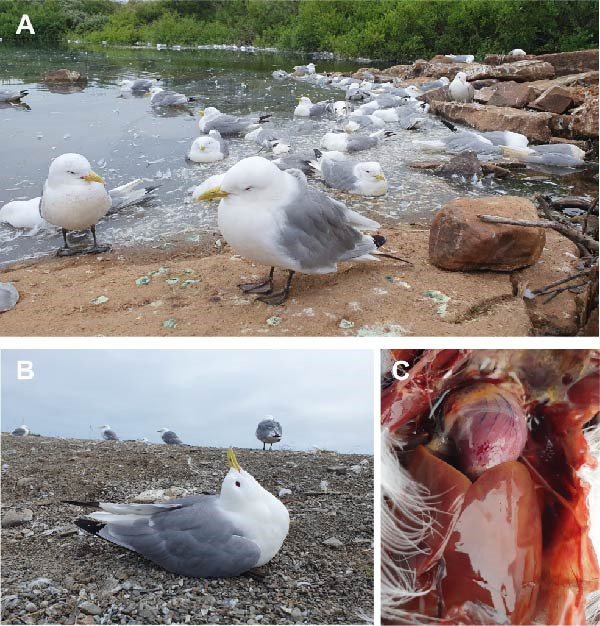
Photos taken during fieldwork at the sampling point at the Storelva River near Ekkerøy, Norway, on July 26, 2023. (A) Kittiwakes suffering from HPAI H5N1, several with squinting eyes and signs of apathy. In the background, dead birds and feathers floating in the water. (B) Kittiwake with opisthotonus (star gazing). (C) Epicardial petechiae and serous fluid in the body cavity and air sacs.

Clinically diseased kittiwakes exhibited general lethargy and severe neurological signs, including disturbed balance, trembling, falling over, or swimming in circles. Observations included opisthotonus, or head twitching, squinting eyes, and unilateral wing lameness, progressing to paralysis, paresis, recumbency, and death (Figure 2A,B, Supporting Information 2: Video).

### 3.3. Necropsy

The necropsied kittiwakes, six females and four males, were adults. Two were in good condition, four were in moderate condition, and four were emaciated (Supporting Information 1: Appendix 1 Table S3). Internal inspection revealed ample amounts of serous fluid in the body cavity and air sacs of all birds. Large areas of necrosis and hemorrhages in the pancreas, epicardial petechiae, and splenomegaly were observed (Figure 2C).

### 3.4. Histopathology

**Figure 3:**
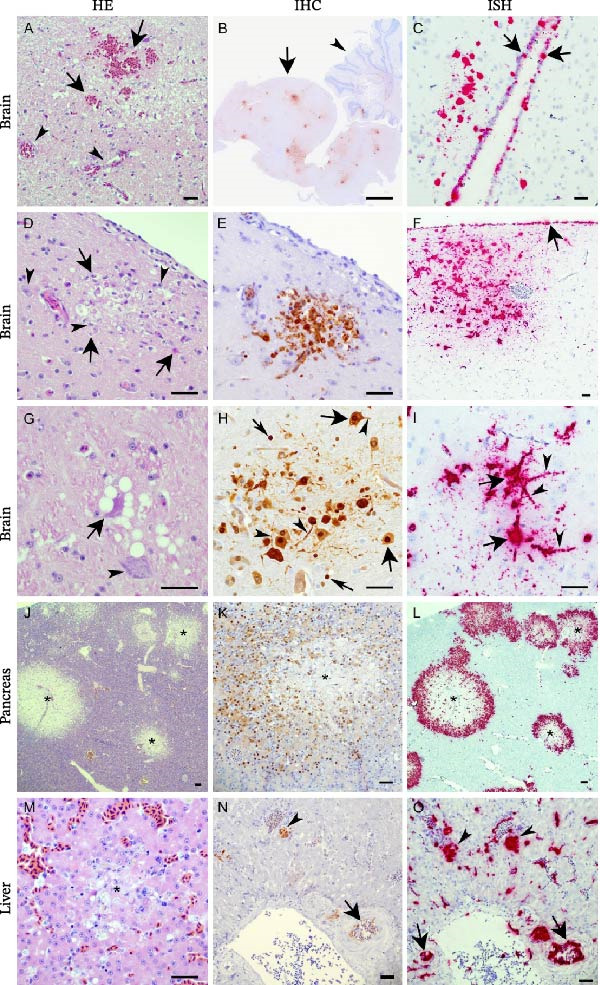
Histopathology and tissue expression of influenza A virus protein and RNA in organs from deceased kittiwakes. Brain (A–I): Hematoxylin and eosin (HE) sections (A, D, and G) demonstrated hemorrhages (A, arrows) and vacuolization of neuropil. Intact blood vessels (A, arrowheads). Necrosis with karyorrhexis and pyknosis (D, arrows) next to viable neurons and neuroglia (D, arrowhead). Single‐cell necrosis with shrunken hyper‐eosinophilic neuron and vacuolized neuropil (G, arrow) and viable neuron (G, arrowhead). Immunohistochemical detection of influenza A nucleoprotein (NP) (B, E, H, brown labeling) was observed multifocally in both the cerebrum (B, arrow) and cerebellum (B, arrowhead). Positive labeling for influenza A NP (E) in the area of necrosis is shown in the serial section (D). Both neurons (H, broad arrows) and neuroglia (H, narrow arrows) were labeled, in both cell nuclei and cytoplasm. Note that the cytoplasm of both the cell body and dendrites was labeled (H, arrowheads). In situ hybridization of influenza A virus RNAscope (C, F, I, red labeling) was detected multifocally in the cerebellum and cerebrum in both neurons, neuroglia, and ependyma (C, arrows), and meninges (F, arrow). Both the cell body (I, arrows) and dendrites (I, arrowheads) of neurons were labeled. Pancreas (J–L): HE‐stained sections of pancreas demonstrated multifocal necroses (J,  ^∗^). Labeling for influenza A virus NP (K) and influenza A virus RNA (L) was detected adjacent to the necrotic ( ^∗^) foci in the pancreas. Liver (M–O): HE‐stained sections of liver demonstrated areas of necrosis (M,  ^∗^) with hypereosinophilic hepatocytes and karyorrhectic and pyknotic nuclei. Immunohistochemical labeling for influenza A NP (K, brown labeling) was observed in both hepatocytes (N, arrowhead) and endothelial cells (N, arrow) of blood vessels. In situ hybridization demonstrated a more widespread labeling of hepatocytes (O, arrowheads), endothelial cells of blood vessels (O, arrow), and endothelial cells of hepatic sinusoids. Scale bars of (A), (C–I) indicate 200 µm, while the scale bar of (B) indicates 1000 µm.

All 10 birds displayed mild to moderate histopathological changes in the brain, affecting both cerebrum and cerebellum, with multifocal vascular damage, hemorrhages, vacuolization of neuropil, and multifocal necroses (Figure 3A–I, Supporting Information 1: Appendix 1 Table S4). This varied from single‐cell degeneration and necrosis of ependymal cells, neurons, and neuroglia to large areas of necrosis. Two birds had multifocal mononuclear meningitis. Apart from this, inflammatory cells were not observed. All the birds had multifocal hemorrhages and moderate‐to‐severe and multifocal‐to‐diffuse necroses in the pancreas (Figure 3J–L, Supporting Information 1: Appendix 1 Table S4). In the liver, there were mild to moderate multifocal necroses in all but one bird (Figure 3M–O). No pathology was observed in the myocardium, except in one bird, which had focal subendocardial hemorrhages. No specific findings were observed in the lung, spleen, proventriculus, or kidney. Intralesional agents such as bacteria, parasites, or fungi were not observed.

### 3.5. Immunohistochemical Detection of Influenza A Virus Nucleoprotein (NP)

IHC staining for influenza A virus NP in brain tissues revealed positive labeling multifocally in and adjacent to necrotic areas, in a large number of individual neurons, glial cells, and ependymal cells, and in a lower number of meningeal and vascular endothelial cells (Figure 3B,E,H). Evaluation of the pancreas revealed a positive signal multifocally, and commonly adjacent to the necrotic foci (Figure 3K). In the liver, a positive signal was present in hepatocytes and endothelial cells of sinusoidal capillaries and larger blood vessels (Figure 3N). No signal was detected in the lung, spleen, or heart muscle.

### 3.6. In Situ Detection of Influenza A Viral RNA

RNA‐ISH of brain, pancreas, liver, and spleen tissues from selected birds revealed signals of various intensities, indicating the presence of viral RNA (Figure 3C,F,I,L,O). The labeling was strong in the cerebrum, cerebellum, and pancreas, aligning with the IHC, albeit stronger and more widespread in the vascular endothelium. Labeling of the liver varied across individuals, from sparse pinpoint signal to strong, multifocal labeling of hepatocytes and endothelial cells throughout the tissue. Spleens from two birds were examined for viral RNA, one displaying a weak positive signal multifocally and the other being negative.

### 3.7. Distribution of Virus Receptors

**Figure 4:**
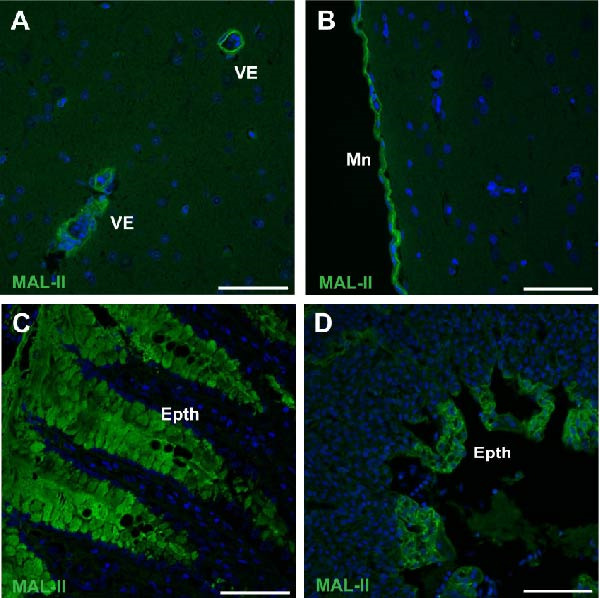
Lectin staining of presumptive avian influenza virus receptors. MAL‐II (green) was used to label α‐2,3‐linked sialic acids in the brain (A, B), lung (C), and proventriculus (D) of deceased kittiwakes. Nuclei are labeled by Hoechst 33342 (blue). Epth, epithelium; Mn, meninges; VE, vascular endothelium. Scale bars indicate 50 µm.

MAL‐II labeled the surface of vascular endothelial cells in all examined kittiwake organs, suggesting the presence of α‐2,3‐linked sialic acids (SAs) that function as AIV receptors. Labeling in the brain was sparse and confined to vascular, ependymal, and meningeal structures, but no convincing signal was observed in neurons or glial cells (Figure 4A,B). Labeling was present in the mucus‐secreting epithelium of the proventriculus and the lung epithelium (Figure 4C,D).

### 3.8. Virological Investigation

**Figure 5:**
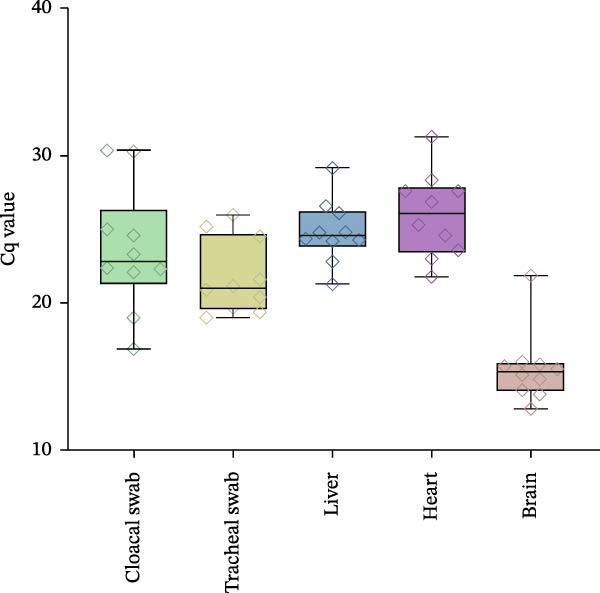
Box‐and‐whisker plot showing the quantification cycle (Cq) values from rRT‐PCR for influenza A virus M gene performed on swab and tissue samples collected from deceased kittiwakes (*n* = 10), Ekkerøy, Norway, July 2023. The central line represents the median, with the whiskers indicating the range of maximum and minimum Cq values. The individual measurements are indicated by diamonds.

High virus levels were detected in swab and tissue samples from all individuals by rRT‐PCR. The highest mean level, indicated by the quantification cycle (Cq) values, was detected in the brain (Cq 15.2), followed by tracheal swabs (Cq 21.8), cloacal swabs (Cq 23.6), liver (Cq 24.8), and heart (Cq 26.0) (Figure 5).

**Figure 6:**
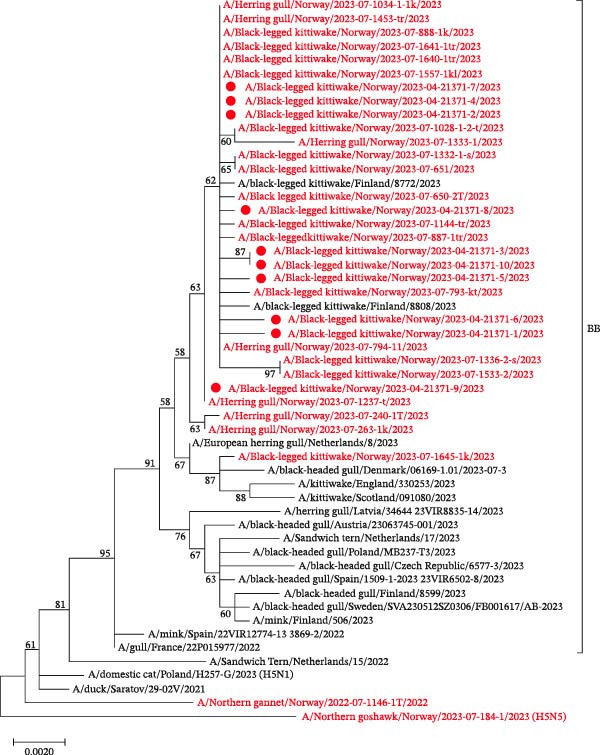
Midpoint rooted phylogenetic tree of the hemagglutinin gene from 10 HPAI H5N1 viruses identified in dead black‐legged kittiwakes from Ekkerøy, Norway, in 2023 (red dots), including contemporary genotype BB viruses from Norway (red) and Europe (black). Bootstrap values >50 are shown.

HPAI H5N1 clade 2.3.4.4b viruses with the hemagglutinin (HA) cleavage site PLREKRRKR/GLF were confirmed in all 10 birds, using rRT‐PCR and WGS. No evidence of co‐infection with other AIV subtypes was detected by WGS. Similarity searches in GISAID BLASTn showed that the viruses from kittiwakes at Ekkerøy shared 99%–100% identity with contemporaneous genotype BB viruses from Europe. The phylogenetic analysis showed that the viruses clustered together and were highly similar to other viruses in gulls circulating in Norway, as well as Europe, during the spring and summer of 2023 (Figure 6, Supporting Information 1: Appendix 1 Figure S2 A–G, Supporting Information 3: Appendix 2).

Mutation analyses showed that the viruses detected in Ekkerøy kittiwakes remained mainly bird‐adapted and had a typical profile of the BB genotype, including NP‐Y52N and M2‐A30S (Supporting Information 4: Appendix 3). Key substitutions in the HA or polymerase proteins previously associated with increased mammalian adaptation were not identified. No neuraminidase mutations associated with oseltamivir resistance were present.

### 3.9. Management and Outbreak Response

During the outbreak, the municipality organized the removal and registration of carcasses. People handling the birds used disposable gloves, coveralls, goggles, and FFP3 masks. A sealed container with top‐loading access was installed in the area to enable hygienic and secure disposal of bird carcasses. Following necropsy and sample collection, the remains were deposited in the container. Once full, it was transported to a certified incineration facility in compliance with environmental and biosecurity regulations.

Compared to Ekkerøy, the observed mortality in the neighboring kittiwake breeding colony at Hornøya in Vardø municipality was low (observation, Tone Reiertsen). To prevent disturbance of vulnerable birds and further viral spread, movement restrictions to Hornøya and two other nature reserves were prioritized and implemented on July 27, 2023 [19]. At Ekkerøy, signs providing information about the outbreak and discouraging traffic in the area were posted. No restrictions were imposed on livestock grazing in the area.

## 4. Discussion

This study documents mass mortality in kittiwakes caused by HPAI H5N1 and enhances the understanding of the disease, pathology, and AIV receptor distribution in this species. Our estimate of the Ekkerøy breeding population size prior to the outbreak is associated with uncertainty, as it does not take into account immature individuals and “floaters” (i.e., sexually mature birds that do not breed). This implies that Ekkerøy’s total population in 2023 might have included as many as 30,000 kittiwakes [20]. Hence, with 15,235 reported dead birds and an assumption of a significant number of unreported cases, HPAI is likely to have caused a colony population reduction of at least 50%.

Seabird populations grow slowly due to their low reproduction rates and the years they spend at sea as immatures before breeding. Acute declines can severely affect future population status [21]. The recovery potential depends on long‐term trends in the population. Declining populations rarely recover, and the time to extinction is usually shortened [22]. The HPAI outbreak may therefore have dramatic long‐term impacts on the kittiwake population in affected areas.

Clinical presentation and pathological lesions of HPAI vary by species and viral strain [23]. The clinical signs of the infected kittiwakes indicate a disease prominently affecting the central nervous system (CNS), aligning with the neurotropic nature of the virus [24–32].

A pathological finding commonly described in gulls infected with HPAIV is a subacute disease with lymphoplasmacytic encephalitis and areas of gliosis [26, 27, 33]. This differs from our findings, where deceased kittiwakes demonstrated widespread necrosis in the brain, pancreas, and liver, with no or limited signs of inflammation. This could suggest hematogenous spread of the HPAI H5N1 genotype BB infection with limited time for an inflammatory reaction to take place prior to death, defining it to be a peracute, highly pathogenic, systemic, and fatal disease in kittiwakes. One study detected considerable species variation in the severity of CNS inflammation among wild birds naturally infected with the HPAI H5N1 virus, ranging from severe in swans and Canada geese to undetectable in a herring gull [27]. Experimental inoculation of ducks and wild‐caught laughing gulls (*Larus atricilla*) with the HPAI H5N1 virus produced necrosis in the brain and pancreas of birds that died or were euthanized due to severe illness, corresponding to our findings in kittiwakes [25]. In birds that recovered, mild lesions such as lymphoplasmacytic perivascular encephalitis and heterophilic pancreatitis were demonstrated. As our study only included deceased birds, we do not know if any infected birds recovered from the infection and whether these then would present similar subacute inflammatory lesions.

Aligning with hallmark features of HPAI, the kittiwakes showed signs of widespread systemic infection, with detection of HPAI H5N1 viral RNA in the brain, liver, and heart, in addition to the trachea and cloaca. By the time of death, the highest viral levels were detected in the brain, supported by low Cq values and extensive labeling by IHC and RNA‐ISH. This corresponded with the histopathological lesions being most evident in this organ. Widespread histopathological lesions, in addition to extensive IHC and RNAscope labeling, indicate high virus levels also in the pancreas. We did not perform rRT‐PCR on pancreatic samples. However, based on the extensive pathology in this organ, we recommend that rRT‐PCR of pancreatic samples be included in future studies.

Virus attachment to cells is a key factor for determining viral tropism, with AIV HA preferentially binding α2,3‐linked SAs [34]. HPAIV replication in endothelial cells is common in chickens and swans, but less so in ducks and mammals [35]. Interestingly, α2,3‐linked SAs were prominently detected on vascular endothelial cells in kittiwakes. Vascular damage with hemorrhages and endothelial expression of viral RNA and antigen were observed in the brain and pancreas. This indicates that kittiwake endothelial cells are susceptible to HPAIV, aligning with the hypothesis of hematogenous spread.

Despite the strong expression of influenza A viral RNA and NP in the brain, neurons were not labeled by MAL‐II, suggesting that H5N1 targets structures in neuronal cell types that do not overlap with MAL‐II binding sites (Sia‐α2,3‐Galβ1‐GalNAc). Gangliosides are the main carriers of both α2,3‐ and α2,6‐linked SAs in the CNS and support the attachment of different influenza A virus strains [36, 37]. While comparative studies of noninfected kittiwake tissues would be interesting, samples from healthy kittiwakes were not feasible due to their endangered status in Norway. The entry point of the virus could not be determined in our study. Like previous observations in herring gulls, laughing gulls, and ring‐billed gulls (*Larus delawarensis*) [38], the mucus‐secreting epithelium of kittiwake proventriculus and lung epithelium expressed MAL‐II targeted SAs.

The genetic similarity between the HPAI H5N1 viruses detected in our study and genotype BB viruses circulating in Europe during spring 2023 suggests introduction to breeding colonies in Northern Norway by migratory birds from Europe. The viruses in kittiwakes were highly similar to each other and clustered with the BB genotype viruses detected in other gull species in Norway, indicating that the outbreak at Ekkerøy was part of a larger epizootic. The temporal succession of positive cases within Northern Norway supports a gradual move of the virus from southwest to northeast. We cannot exclude virus introduction to kittiwakes during their pelagic phase in the Atlantic Ocean when they mix with other seabirds. However, we found no data to support this, and such an introduction would have required a longer presymptomatic phase of infection. Two HPAIV detections in kittiwakes in Finland in July 2023 clustered among the viruses from gulls in Northern Norway, indicating the same origin. Another virus from Southern Norway in July 2023 (A/Black‐legged_kittiwake/Norway/2023‐07‐1645‐1k/2023) clustered with HPAI H5N1 viruses detected in gulls in Denmark, England, and Scotland in the summer of 2023 and probably represents a separate introduction from the North Sea region.

Due to the consistent clinical signs, pathological findings, and detection of high levels of HPAI H5N1 infection in all examined birds, together with the absence of intralesional infectious agents (such as bacteria) and inflammation, an extensive differential diagnostic workup was not performed in this outbreak. Notably, WGS did not identify any evidence of mixed AIV infection.

Kittiwake mortality varied considerably between colonies, for instance, between Ekkerøy and Hornøya. A similar pattern was seen in tern colonies in the German Wadden Sea area in the summer of 2022, with some sites experiencing up to 40% mortality and others mostly spared [39]. Factors influencing mortality numbers include host susceptibility, colony size, species diversity, behavioral changes, bird movement patterns, immune status, timing, magnitude of virus introduction, environmental factors, and varying recording [40–42]. Understanding transmission within and between colonies requires wildlife community surveillance focusing on all interacting species.

Several factors likely contributed to the severity of the HPAI outbreak in kittiwakes. Due to the pelagic lifestyle of kittiwakes, preexisting immunity to H5 is assumed to have been low prior to the epizootic in 2023 [9, 12]. Virus properties, specifically the genetic constellation of the BB genotype, also likely enhanced virus replication in gulls [43]. Kittiwakes nest closely together, facilitating virus transmission. The outbreak at Ekkerøy coincided with a high number of susceptible chicks and adults with a reduced body condition toward the end of the breeding period that could have lowered their resistance and resilience to infection. At the Storelva River near Ekkerøy, numerous infected carcasses, feathers, and feces combined with low water levels likely promoted virus spread through freshwater. Environmental samples could have provided more information and should be obtained in future outbreaks.

The viruses detected in kittiwakes at Ekkerøy were avian‐adapted with mutation profiles similar to other BB genotype viruses, thus not indicating elevated risk for mammal adaptation. However, a few mutations that could increase the zoonotic risk were present. NP‐Y52N associated with evasion of human BTN3A3 restriction was identified [44].

Mitigation measures can be implemented to reduce HPAI spread and protect vulnerable wild bird populations. One measure is removal of carcasses, but this must be balanced against the risk of disturbing the remaining birds in the colony. The effect of carcass removal is difficult to measure. A study of HPAI‐affected sandwich tern (*Thalasseus sandvicensis*) colonies found that carcass removal reduced adult mortality by an average of 15% [45]. To keep the infection pressure in the environment low and reduce spillover to other birds and mammals, carcass removal should start early in an outbreak and be performed by trained personnel using personal protective equipment. Vadsø has only 5800 inhabitants, a long coastline, and minimal livestock farming. The high number of bird carcasses at Ekkerøy was challenging to handle due to limited staff in this remote area, coinciding with summer vacation, underlining the need for joint effort in extreme situations.

Vaccination of endangered bird species may be considered as a preventive measure for conservation in extreme cases [46]. However, for kittiwakes, oral or bait‐based delivery is not feasible due to their feeding behavior, and intramuscular administration poses logistical challenges in large seabird colonies. Additionally, vaccine efficacy may vary according to species and circulating viral strain.

Early detection of viruses with increased ability to infect mammals is crucial for pandemic preparedness. Management of HPAI mass mortalities in wildlife, therefore, requires a multisectoral One Health approach with clearly defined organizational roles. Active cross‐species surveillance to detect potential spillover was not performed during the outbreak in 2023 but is recommended during future wildlife outbreaks, with a primary focus on scavengers, predators, and marine mammals. Passive surveillance for avian influenza was ongoing but with limited samples from the area, and some cases in wildlife may have gone undetected. White‐tailed eagles (*Haliaeetus albicilla*) were observed at Ekkerøy during the outbreak. However, no disease or mortality was noted, suggesting that they were less susceptible to the BB genotype [47].

After it became known from the USA in March 2024 that ruminants can be infected with HPAIV, we performed post‐outbreak surveillance in sheep that had grazed among sick and dead kittiwakes. H5 antibodies were detected in one sheep 11 months after the outbreak in kittiwakes, indicating that spillover to mammals had occurred rarely [48].

Humans were followed up according to the existing routines recommended by the Norwegian Institute of Public Health at the time. These guidelines stated that the municipal doctor should consider offering postexposure prophylaxis with oseltamivir and recommend PCR testing if clinical symptoms developed within 14 days of exposure. No such cases with clinical symptoms were reported in connection with the outbreak. Serological testing was not an option at the time.

Our study does not determine to what extent kittiwakes survived infection and may have gained immunity. Studies that can provide such data may be of great value for projecting the vulnerability of this and other kittiwake colonies to future HPAI H5Nx outbreaks.

## 5. Conclusions

We recommend further monitoring of the long‐term impact of HPAI on the kittiwake populations. HPAI outbreaks in seabird colonies need awareness and must be mitigated using a multisectoral One Health approach to protect endangered wildlife species, mammals, and public health.

## Author Contributions

Conceptualization, funding acquisition: Grim Rømo, Caroline Piercey Åkesson, Lars Austbø, Johanna Hol Fosse, and Ragnhild Tønnessen. Methodology, investigation: Grim Rømo (field work, epidemiology), Caroline Piercey Åkesson (pathology), Lars Austbø (PCR investigations), Johanna Hol Fosse (receptor studies), Johan Åkerstedt (epidemiology, geographic mapping), Maryam Saghafian (staining techniques), Cathrine Arnason Bøe (whole genome sequencing, genotyping), Olav Hungnes and Ragnhild Tønnessen (phylogeny, mutation screening), and Tone Kristin Reiertsen (mortality/population estimation). Writing – original draft, writing – review and editing: All authors. Project administration: Grim Rømo and Ragnhild Tønnessen. Supervision: Ragnhild Tønnessen and Caroline Piercey Åkesson.

## Funding

The work presented in this paper was funded by the Norwegian Veterinary Institute.

## Disclosure

The work presented in this article has been published as a preprint [49].

## Ethics Statement

Carcass handling and disposal procedures followed national regulations and guidelines, and no specific permits were required for this study.

## Conflicts of Interest

The authors declare no conflicts of interest.

## Supporting Information

Additional supporting information can be found online in the Supporting Information section.

## Supporting information


**Supporting Information 1** Appendix 1: Detailed methods and additional results. Table S1: Number of dead birds reported by the municipalities in Troms and Finnmark County, Norway, between July 28 and August 28, 2023, due to the HPAI H5N1 outbreak in kittiwakes (*Rissa tridactyla*) in Northern Norway. Table S2: Counts of apparently occupied nests during the outbreak, used for the estimation of the number of breeding pairs of black‐legged kittiwakes at the Ekkerøy colony, Norway, in 2023. Table S3: Individual data and pectoral muscle condition score (PMCS) of 10 black‐legged kittiwakes that died from HPAI H5N1 and were necropsied at Storelva, Norway, in July 2023. Table S4: Score of histopathological examination: Hematoxylin and eosin (HE)‐stained slides of organs from 10 black‐legged kittiwakes that died from HPAI H5N1 at Ekkerøy, Norway, in 2023, were examined and scored semiquantitatively based on histopathological observations. Figure S1: A photo segment of the kittiwake cliff on Ekkerøy from July 2023, showcasing the image resolution. Apparently, occupied nests were counted from several such pictures to estimate the size of the breeding population in 2023. Photo: Knut Sverre Horn. Figure S2: Midpoint rooted phylogenetic trees showing the genetic relationship between 10 HPAI H5N1 viruses identified in dead black‐legged kittiwakes from Ekkerøy, Norway, in 2023 (red dots), and contemporary viruses from Norway (red) and Europe (black), including the coding part of the gene segments (A) PB2, (B) PB1, (C) PA, (D) NP, (E) NA, (F) M, and (G) NS.

**Supporting Information 2** Video clip. Video footage from a highly pathogenic avian influenza (HPAI) H5N1 outbreak among black‐legged kittiwakes (*Rissa tridactyla*) in Vadsø, Norway, in July 2023. The video shows mass mortality and birds exhibiting clinical signs of neurological dysfunction.

**Supporting Information 3** Appendix 2: Data included in the phylogenetic analyses.

**Supporting Information 4** Appendix 3: Table showing markers which might indicate mammalian adaptation identified using FluMut in whole genome sequenced HPAI H5N1 clade 2.3.4.4b genotype BB viruses from 10 black‐legged kittiwakes at Ekkerøy in Norway, 2023.

## Data Availability

All data are available in the article’s Supporting Information, except for data on virus detections, which are available upon request from the Norwegian Veterinary Institute.

## References

[1] EFSA (European Food Safety Authority) , ECDC (European Centre for Disease Prevention and Control) , and EURL (European Union Reference Laboratory for Avian Influenza) , et al.Avian Influenza Overview March–April 2023, EFSA Journal. (2023) 21, no. 6, e08039. 37293570 10.2903/j.efsa.2023.8039 PMC10245295

[2] Banyard A. C. , Bennison A. , and Byrne A. M. P. , et al.Detection and Spread of High Pathogenicity Avian Influenza Virus H5N1 in the Antarctic Region, Nature Communications. (2024) 15, no. 1, 10.1038/s41467-024-51490-8, 7433. PMC11372179 39227574

[3] Jimenez-Bluhm P. , Siegers J. Y. , and Tan S. , et al.Detection and Phylogenetic Analysis of Highly Pathogenic A/H5N1 Avian Influenza Clade 2.3.4.4b Virus in Chile, 2022, Emerging Microbes & Infections. (2023) 12, no. 2, 10.1080/22221751.2023.2220569, 2220569. 37254689 PMC10283444

[4] Fusaro A. , Zecchin B. , and Giussani E. , et al.High Pathogenic Avian Influenza A(H5) Viruses of Clade 2.3.4.4b in Europe—Why Trends of Virus Evolution Are More Difficult to Predict, Virus Evolution. (2024) 10, no. 1, 10.1093/ve/veae027, veae027. 38699215 PMC11065109

[5] Adlhoch C. , Fusaro A. , and Gonzales J. L. , et al.Avian influenza overview April – June 2023, EFSA Journal. (2023) 2023, Report No.: 1831-4732. 10.2903/j.efsa.2023.8191 PMC10358191 37485254

[6] Munster V. J. , Baas C. , and Lexmond P. , et al.Spatial, Temporal, and Species Variation in Prevalence of Influenza A Viruses in Wild Migratory Birds, PLoS Pathogens. (2007) 3, no. 5, 10.1371/journal.ppat.0030061, 2-s2.0-34249726105, e61. 17500589 PMC1876497

[7] Stokke B. G. , Dale S. , Jacobsen K.-O. , Lislevand T. , Solvang R. , and Strøm H. , Evaluation of the Population of Kittiwakes, *Rissa tridactyla* Norway (Vurdering av krykkje *Rissa tridactyla* for Norge). *Rødlista for arter 2021* , 2021, Artsdatabanken.

[8] Systad G. and Vang R. , SEAPOP - Last Observation per Locality in Breeding Season, 2024, Norwegian Institute for Nature Research.

[9] Toennessen R. , Germundsson A. , and Jonassen C. M. , et al.Virological and Serological Surveillance for Type A Influenza in the Black-Legged Kittiwake (*Rissa tridactyla*), Virology Journal. (2011) 8, 21. 21241499 10.1186/1743-422X-8-21 PMC3032712

[10] Hall J. S. , Teslaa J. L. , and Nashold S. W. , et al.Evolution of a Reassortant North American Gull Influenza Virus Lineage: Drift, Shift and Stability, Virology Journal. (2013) 10, 179. 23742717 10.1186/1743-422X-10-179 PMC3706275

[11] Lee M. M. , Jaspers V. L. B. , and Gabrielsen G. W. , et al.Evidence of Avian Influenza Virus in Seabirds Breeding on a Norwegian High-Arctic Archipelago, BMC Veterinary Research. (2020) 16, no. 1, 10.1186/s12917-020-2265-2, 48. 32028933 PMC7006154

[12] Greco F. , Ravenswater H. M. , and Ruiz-Raya F. , et al.Asymptomatic Infection and Antibody Prevalence to Co-Occurring Avian Influenza Viruses Vary Substantially Between Sympatric Seabird Species Following H5N1 Outbreaks, Scientific Reports. (2025) 15, no. 1, 10.1038/s41598-025-85152-6, 1435. 39789128 PMC11718005

[13] McPhail G. M. , Collins S. M. , and Burt T. V. , et al.Geographic, Ecological, and Temporal Patterns of Seabird Mortality During the 2022 HPAI H5N1 Outbreak on the Island of Newfoundland, Canadian Journal of Zoology. (2024) 10.1101/2024.01.17.575746.

[14] Agüero M. , Monne I. , and Sánchez A. , et al.Highly Pathogenic Avian Influenza A(H5N1) Virus Infection in Farmed Minks, Spain, October 2022, Euro Surveillance. (2023) 28, no. 3. 10.2807/1560-7917.ES.2023.28.3.2300001 PMC9853945 36695488

[15] Lindh E. , Lounela H. , and Ikonen N. , et al.Highly Pathogenic Avian Influenza A(H5N1) Virus Infection on Multiple Fur Farms in the South and Central Ostrobothnia Regions of Finland, July 2023, Euro Surveillance. (2023) 28, no. 31. 10.2807/1560-7917.ES.2023.28.31.2300400 PMC10401912 37535475

[16] Leguia M. , Garcia-Glaessner A. , and Muñoz-Saavedra B. , et al.Highly Pathogenic Avian Influenza A (H5N1) in Marine Mammals and Seabirds in Peru, Nature Communications. (2023) 14, no. 1, 10.1038/s41467-023-41182-0, 5489. PMC10484921 37679333

[17] Burrough E. , Magstadt D. , and Petersen B. , et al.Highly Pathogenic Avian Influenza A(H5N1) Clade 2.3.4.4b Virus Infection in Domestic Dairy Cattle and Cats, United States, 2024, Emerging Infectious Diseases. (2024) 30, no. 7, 1335–1343, 10.3201/eid3007.240508. 38683888 PMC11210653

[18] EFSA (European Food Safety Authority) , ECDC (European Centre for Disease Prevention and Control) , and EURL (European Union Reference Laboratory for Avian Influenza) , et al.Scientific Report: Avian Influenza Overview September–December 2024, 2025, EFSA. 10.2903/j.efsa.2025.9204 PMC11719707 39802641

[19] Ministry of Agriculture and Food , Regulations on Temporary Traffic Bans to Prevent the Spread of Highly Pathogenic Avian Influenza to Wild Birds, 2023, Norwegian Food Safety Authority.

[20] Kokko H. , Harris M. P. , and Wanless S. , Competition for Breeding Sites and Site-Dependent Population Regulation in a Highly Colonial Seabird, the Common Guillemot *Uria aalge* , Journal of Animal Ecology. (2004) 73, no. 2, 367–376, 10.1111/j.0021-8790.2004.00813.x, 2-s2.0-1542438549.

[21] Erikstad K. E. , Fauchald P. , Tveraa T. , and Steen H. , On the Cost of Reproduction in Long-Lived Birds: The Influence of Environmental Variability, Ecology. (1998) 79, no. 5, 1781–1788, 10.1890/0012-9658(1998)079[1781:OTCORI]2.0.CO;2, 2-s2.0-0031853276.

[22] Reiertsen T. K. , Erikstad K. E. , and Johansen M. K. , et al.Effects of Acute Population Reductions in Seabirds Linked to Lofoten, Vesterålen and the Barents Sea [Effekter av akutte bestandsreduksjoner hos sjøfugl knyttet til Lofoten, Vesterålen og Barentshavet], 2019.

[23] Swayne D. E. , Understanding the Complex Pathobiology of High Pathogenicity Avian Influenza Viruses in Birds, Avian Diseases. (2007) 51, no. 1 Suppl, 242–249, 10.1637/7763-110706-REGR.1. 17494560

[24] Breithaupt A. , Kalthoff D. , Dale J. , Bairlein F. , Beer M. , and Teifke J. P. , Neurotropism in Blackcaps (*Sylvia atricapilla*) and Red-Billed Queleas (*Quelea quelea*) after Highly Pathogenic Avian Influenza Virus H5N1 Infection, Veterinary Pathology. (2011) 48, no. 5, 924–932, 10.1177/0300985810386467, 2-s2.0-80051991361. 20974871

[25] Brown J. D. , Stallknecht D. E. , Beck J. R. , Suarez D. L. , and Swayne D. E. , Susceptibility of North American Ducks and Gulls to H5N1 Highly Pathogenic Avian Influenza Viruses, Emerging Infectious Diseases. (2006) 12, no. 11, 1663–1670, 10.3201/eid1211.060652, 2-s2.0-33750555945. 17283615 PMC3372354

[26] Brown J. D. , Stallknecht D. E. , and Swayne D. E. , Experimental Infections of Herring Gulls (*Larus argentatus*) With H5N1 Highly Pathogenic Avian Influenza Viruses by Intranasal Inoculation of Virus and Ingestion of Virus-Infected Chicken Meat, Avian Pathology. (2008) 37, no. 4, 393–397, 10.1080/03079450802216595, 2-s2.0-47549101849. 18622855

[27] Bröjer C. , Ågren E. O. , Uhlhorn H. , Bernodt K. , Jansson D. S. , and Gavier-Widén D. , Characterization of Encephalitis in Wild Birds Naturally Infected by Highly Pathogenic Avian Influenza H5N1, Avian Diseases. (2012) 56, no. 1, 144–152, 10.1637/9826-060111-Reg.1, 2-s2.0-84859331586. 22545540

[28] Ellis T. M. , Barry Bousfield R. , and Bissett L. A. , et al.Investigation of Outbreaks of Highly Pathogenic H5N1 Avian Influenza in Waterfowl and Wild Birds in Hong Kong in Late 2002, Avian Pathology. (2004) 33, no. 5, 492–505, 10.1080/03079450400003601, 2-s2.0-4644226853. 15545029

[29] Klopfleisch R. , Werner O. , Mundt E. , Harder T. , and Teifke J. P. , Neurotropism of Highly Pathogenic Avian Influenza Virus A/Chicken/Indonesia/2003 (H5N1) in Experimentally Infected Pigeons (*Columbia livia f. domestica*), Veterinary Pathology. (2006) 43, no. 4, 463–470, 10.1354/vp.43-4-463, 2-s2.0-33746000444. 16846988

[30] Pantin-Jackwood M. J. and Swayne D. E. , Pathogenesis and Pathobiology of Avian Influenza Virus Infection in Birds, Revue Scientifique et Technique de l’OIE. (2009) 28, no. 1, 113–136, 10.20506/rst.28.1.1869. 19618622

[31] Perkins L. E. L. and Swayne D. E. , Susceptibility of Laughing Gulls (*Larus atricilla*) to H5N1 and H5N3 Highly Pathogenic Avian Influenza Viruses, Avian Diseases. (2002) 46, no. 4, 877–885, 10.1637/0005-2086(2002)046[0877:SOLGLA]2.0.CO;2, 2-s2.0-0036816794. 12495048

[32] Szeredi L. , Dán Á. , and Pálmai N. , et al.Tissue Tropism of Highly Pathogenic Avian Influenza Virus Subtype H5N1 in Naturally Infected Mute Swans (*Cygnus Olor*), Domestic Geese (*Anser Anser var. domestica*), Pekin Ducks (*Anas platyrhynchos*) and Mulard Ducks (*Cairina moschata* × *anas platyrhynchos*), Acta Veterinaria Hungarica. (2010) 58, no. 1, 133–145, 10.1556/avet.58.2010.1.14, 2-s2.0-77954630655. 20514747

[33] Gulyaeva M. A. , Sharshov K. A. , Zaykovskaia A. V. , Shestopalova L. V. , and Shestopalov A. M. , Experimental Infection and Pathology of Clade 2.2 H5N1 Virus in Gulls, Journal of Veterinary Science. (2016) 17, no. 2, 179–188, 10.4142/jvs.2016.17.2.179, 2-s2.0-85009186255. 26243601 PMC4921666

[34] Long J. S. , Mistry B. , Haslam S. M. , and Barclay W. S. , Host and Viral Determinants of Influenza A Virus Species Specificity, Nature Reviews Microbiology. (2019) 17, no. 2, 67–81. 30487536 10.1038/s41579-018-0115-z

[35] Kuiken T. , van den Brand J. , van Riel D. , Pantin-Jackwood M. , and Swayne D. E. , Comparative Pathology of Select Agent Influenza A Virus Infections, Veterinary Pathology. (2010) 47, no. 5, 893–914. 20682805 10.1177/0300985810378651

[36] Krengel U. and Bousquet P. A. , Molecular Recognition of Gangliosides and Their Potential for Cancer Immunotherapies, Frontiers in Immunology. (2014) 5, 10.3389/fimmu.2014.00325, 2-s2.0-84905647126, 325. 25101077 PMC4104838

[37] Suzuki Y. , Nagao Y. , and Kato H. , et al.Human Influenza A Virus Hemagglutinin Distinguishes Sialyloligosaccharides in Membrane-Associated Gangliosides as Its Receptor Which Mediates the Adsorption and Fusion Processes of Virus Infection. Specificity for Oligosaccharides and Sialic Acids and the Sequence to which Sialic Acid Is Attached, The Journal of Biological Chemistry. (1986) 261, no. 36, 17057–17061. 3782153

[38] França M. , Stallknecht D. E. , and Howerth E. W. , Expression and Distribution of Sialic Acid Influenza Virus Receptors in Wild Birds, Avian Pathology. (2013) 42, no. 1, 60–71. 23391183 10.1080/03079457.2012.759176 PMC3573863

[39] Pohlmann A. , Stejskal O. , and King J. , et al.Mass Mortality Among Colony-Breeding Seabirds in the German Wadden Sea in 2022 due to Distinct Genotypes of HPAIV H5N1 Clade 2.3.4.4b, Journal of General Virology. (2023) 104, no. 4, 10.1099/jgv.0.001834. 37014781

[40] Caliendo V. , Kleyheeg E. , and Beerens N. , et al.Effect of 2020-21 and 2021-22 Highly Pathogenic Avian Influenza H5 Epidemics on Wild Birds, the Netherlands, Emerging Infectious Diseases. (2024) 30, no. 1, 50–57. 38040665 10.3201/eid3001.230970 PMC10756359

[41] Falchieri M. , Reid S. M. , and Ross C. S. , et al.Shift in HPAI Infection Dynamics Causes Significant Losses in Seabird Populations Across Great Britain, Veterinary Record. (2022) 191, no. 7, 294–296, 10.1002/vetr.2311. 36205958

[42] Jeglinski J. W. E. , Lane J. V. , and Votier S. C. , et al.HPAIV Outbreak Triggers Short-Term Colony Connectivity in a Seabird Metapopulation, Scientific Reports. (2024) 14, no. 1, 10.1038/s41598-024-53550-x, 3126. 38326368 PMC10850054

[43] Tønnessen R. , Hauge A. G. , Hansen E. F. , Rimstad E. , and Jonassen C. M. , Host Restrictions of Avian Influenza Viruses: In Silico Analysis of H13 and H16 Specific Signatures in the Internal Proteins, PLoS One. (2013) 8, no. 4, 10.1371/journal.pone.0063270, 2-s2.0-84876961057, e63270. 23646204 PMC3639990

[44] Pinto R. M. , Bakshi S. , and Lytras S. , et al.BTN3A3 Evasion Promotes the Zoonotic Potential of Influenza A Viruses, Nature. (2023) 619, no. 7969, 338–347, 10.1038/s41586-023-06261-8.37380775

[45] Knief U. , Bregnballe T. , and Alfarwi I. , et al.Highly Pathogenic Avian Influenza Causes Mass Mortality in Sandwich Tern *Thalasseus sandvicensis* Breeding Colonies Across North-Western Europe, Bird Conservation International. (2024) 34, 10.1017/S0959270923000400, e6.

[46] Lejeune M. , Tornos J. , and Bralet T. , et al.Vaccination Against H5 HP Influenza Virus Leads to Persistent Immune Response in Wild King Penguins, bioRxiv. 17, 10.1101/2025.09.06.674613. PMC12886961 41663358

[47] Bøe C. A. , Fiskebeck E. M. L. Z. , and Reiten M. R. , et al.Emergence of Highly Pathogenic Avian Influenza Viruses H5N1 and H5N5 in White-Tailed Eagles, 2021–2023, Journal of General Virology. (2024) 105, no. 11, 10.1099/jgv.0.002035. PMC11529892 39485726

[48] Fosse J. H. , Rømo G. , and Bonfante F. , et al.Detection of Antibodies Specific to H5 Avian Influenza Virus in a Sheep in Norway, 11 Months After an Outbreak of Highly Pathogenic Avian Influenza in a Nearby Seabird Colony, Influenza and Other Respiratory Viruses. (2025) 19, no. 11, 10.1111/irv.70182, e70182. 41254898 PMC12626899

[49] Rømo G. , Åkesson C. P. , and Reiertsen T. K. , et al.Highly Pathogenic Avian Influenza A(H5N1) Caused Mass Death Among Black-Legged Kittiwakes (*Rissa tridactyla*) in Norway, 2023, bioRxiv. (2023) 10.1101/2025.05.23.655725. PMC12917261 41725838

