# Supplementary material for: Highly Pathogenic Avian Influenza A (H5N1) Caused Mass Death Among Black‐Legged Kittiwakes (Rissa tridactyla) in Norway, 2023
Source: Transbound Emerg Dis. 2026 Feb 18;2026:2963364. doi: 10.1155/tbed/2963364 (PMC12917261; doi:10.1155/tbed/2963364)
Supplement: Supplementary file 1 — Supporting Information 1 Appendix 1: Detailed methods and additional results. Table S1: Number of dead birds reported by the municipalities in Troms and Finnmark County, Norway, between July 28 and August 28, 2023, due to the HPAI H5N1 outbreak in kittiwakes (Rissa tridactyla) in Northern Norway. Table S2: Counts of apparently occupied nests during the outbreak, used for the estimation of the number of breeding pairs of black‐legged kittiwakes at the Ekkerøy colony, Norway, in 2023. Table S3: Individual data and pectoral muscle condition score (PMCS) of 10 black‐legged kittiwakes that died from HPAI H5N1 and were necropsied at Storelva, Norway, in July 2023. Table S4: Score of histopathological examination: Hematoxylin and eosin (HE)‐stained slides of organs from 10 black‐legged kittiwakes that died from HPAI H5N1 at Ekkerøy, Norway, in 2023, were examined and scored semiquantitatively based on histopathological observations. Figure S1: A photo segment of the kittiwake cliff on Ekkerøy from July 2023, showcasing the image resolution. Apparently, occupied nests were counted from several such pictures to estimate the size of the breeding population in 2023. Photo: Knut Sverre Horn. Figure S2: Midpoint rooted phylogenetic trees showing the genetic relationship between 10 HPAI H5N1 viruses identified in dead black‐legged kittiwakes from Ekkerøy, Norway, in 2023 (red dots), and contemporary viruses from Norway (red) and Europe (black), including the coding part of the gene segments (A) PB2, (B) PB1, (C) PA, (D) NP, (E) NA, (F) M, and (G) NS. [file TBED-2026-2963364-s001.pdf]

Highly Pathogenic Avian Influenza A(H5N1) Caused Mass Death among Black-legged Kittiwakes (*Rissa tridactyla*) in Norway, 2023

Appendix 1

Materials and methods

Organizational structure

The Norwegian Food Safety Authority (NFSA) is the administrative authority for the health and welfare of wild and domestic animals in Norway, and responsible for the surveillance programme for HPAI in wild birds. Wildlife managers, ornithologist and citizens notify the NFSA when HPAI is suspected. The NFSA or their collaborators collect swabs from the trachea and cloaca of the birds and ship the samples to the Norwegian Veterinary Institute (NVI) for analysis. NVI is the national reference laboratory for avian influenza and is responsible for planning laboratory investigation and reporting components of the programme. In addition, NVI conducts risk assessments and provides advice on disease prevention and management in case of outbreaks in animals. The Norwegian Institute of Public Health (NIPH) is responsible for influenza in humans, gives advice on public health aspects and is the national reference laboratory for human influenza as well as the WHO National Influenza Centre.

Epidemiological analysis

To provide an epidemiological description of the outbreak in northern Norway, data on HPAI virus (HPAIV) PCR detections in Black-legged Kittiwakes (*Rissa tridactyla*) were obtained from the laboratory reporting system at the NVI. Data were analysed and maps created using R version 4.3.0 (*1*). Data of locations of Kittiwake nests were obtained from the Species Observations System (*2*).

Population estimates

During the outbreak, the colony was documented through photography (Figure 1), and the total number of apparently occupied nests was recorded to estimate the population of Kittiwake breeding pairs present in 2023 (Table 2). Nests containing chicks or adults or both were counted as occupied nest. In addition, nests with dead chicks or adults or empty nests with current years' nesting material and traces of fresh faeces were counted as occupied. Nests with an uncertain status were included in the final estimate.

Necropsy

During fieldwork in July 2023, necropsy and sample collection were performed from ten recently deceased Kittiwakes. The body condition was determined by scoring the pectoral muscle based on photographs in accordance with the description in Franeker J.A. van & C.J. Camphuysen 2007 (*3*). Swabs (COPAN, eSwab) from trachea and cloaca, and tissue from brain, liver and heart (5 x 5 x 5 mm) were collected separately into tubes containing UTM-medium for PCR analyses at NVI. For histopathology, immunohistochemistry, RNAscope- and receptor analyses whole/half brain and approximately 1 x 1 x 0.5 cm large tissue samples

were collected from heart, lung, liver, kidney, spleen, pancreas and proventriculus and placed in 10% buffered formalin.

Histopathology, immunohistochemistry and RNAscope in situ *hybridization*

Tissue samples fixed in 10% buffered formalin, were subsequently dehydrated in ethanol, equilibrated in xylene and embedded in paraffin. For histology, 2-3 µm thick sections were stained with hematoxylin and eosin (HE) and examined by a trained pathologist. In addition, selected tissue sections were subjected to immunohistochemistry; labelled with antibodies against influenza A nucleoprotein (NP) (Anti Influenza A NP, mouse monoclonal IgG1, produced on virus particles from H5N2 strain, Statens Serum Institut). 2-3 µm tissue sections were mounted onto poly-lysine-coated slides, heated to 60 ºC for at least 30 minutes and deparaffinized. The slides were blocked for endogen peroxidase for ten minutes followed by enzyme treatment applying 2 µl proteinase K recombinant PCR Grade in 4 ml TRIS-buffer applied to the sections for 30 minutes at 37 ºC. Subsequent step included blocking with 5 % BSA in TRIS buffer solution for 20 minutes. The primary anti-influenza A NP antibody was diluted 1:2000 for one hour at room temperature, followed by application of the secondary antibody EnVision HRP anti-mouse for 30 minutes at room temperature. Substrate used for visualization was Romulin AEC chromogen, mixing 16 µl solution A, B and C in 2.5 ml Romulin buffer, 15 minutes at room temperature.

Paraffin-embedded tissue sections were used to detect viral sequences through the RNAscope™ *in situ* hybridization (ISH) technique (*4*). RNAscope ISH is an innovative method that allows the visualization of individual RNA molecules in cytological and histological samples at the single-cell level. In our study, we employed a probe that targets the

matrix gene of the Avian Influenza A Virus (AIAV), which is conserved across all influenza A viruses. This probe was obtained from ACD Bio (RNAscope™ Probe - V-InfluenzaA-H5N8-M2M1-C1, Cat. Nr. 1048221-C1), and the slides were stained using the RNAscope™ 2.5 HD Detection Reagents-RED assay (ACD Bio, Cat. Nr. 322360). For the negative control, we used a probe that targets the dihydrodipicolinate reductase (dapB) gene from *Bacillus subtilis* (Cat No. 310043). For the positive control, RNAscope® Probe - Gg-PPIB - *Gallus gallus* peptidylprolyl isomerase B (cyclophilin B) (PPIB) mRNA (Cat No. 453371) was utilized, which serves as a housekeeping gene.

MAL-II lectin staining

To map the distribution of H5N1 virus receptors, the binding pattern of the lectin MAL-II was assessed (*5*). Avian influenza A viruses bind Siaα2–3Galβ1-terminated glycans, with further diversity in binding specificity depending on underlying glycan types and modifications (*6*). The MAL-II lectin specifically binds Siaα2–3Galβ1-3GalNAcs (*7*). Tissue sections from brain, lung and proventriculus (4 µm) were deparaffinized and incubated MAL-II (CliniSciences, France; 40 µg/mL in PBS) for 60 min at room temperature, washed (PBS, 5 min x 2), counterstained with Hoechst 33342 (2 µg/mL, Thermo Fisher Scientific), and mounted in ProLong Glass Antifade mountant (Thermo Fisher Scientific). Confocal images were obtained using a Zeiss Axio LSM710 equipped with a Plan-Apochromat 40x/1.3 NA oil objective and Zen 2.3SP1 software.

Real-time RT-PCR and whole genome sequencing

Real-time RT-PCR (rRT-PCR) for influenza A virus was performed on the collected cloacal and tracheal swabs, as well as liver, heart, and brain tissue samples. The swabs and tissue samples

were pretreated with MagNA Pure External Lysis Buffer (Roche, 06374913001). Approximately 20 mg of tissue was homogenized before total RNA/DNA was extracted from all samples using MagNA Pure 96 automated extractor (Roche) with the MagNA Pure 96 DNA and viral NA LV Kit (Roche, 6374891001). Detection of Influenza A virus was set up using an rRT-PCR assay targeting the conserved M gene (*8*), in accordance with the Manual of standards for diagnostic tests and vaccines (*9*). Subtyping was performed with a specific assay for the HPAI H5 clade 2.3.4.4b (*10*, *11*) together with HA and NA subtype specific rRT-PCR-assays (*12*, *13*). The rRT-PCR for detection and subtyping of Influenza A virus was carried out with OneStep RT-PCR Kit (Qiagen, 210212) and Brilliant III Ultra-Fast QRT-PCR Master Mix (Agilent Technologies, 600884). All analyses were run on AriaMX instruments by Agilent.

Nucleic acids extracted from the brain samples underwent cDNA synthesis, as described by Zhou et al., 2009 and were subjected to whole genome sequencing (*14*). Briefly, extracted nucleic acids (same as for PCR) were employed as template for cDNA synthesis and subsequent PCR using degenerate primers amplifying all 8 segments of the Influenza A virus (Superscript III Platinum one-step quantitative RT-PCR system (Thermo Fisher Scientific, Waltham, MA, USA)). The PCR products were analyzed and quantified on a TapeStation 4200 (Agilent Technologies, Santa Clara, CA, USA). DNA libraries were prepared using Illumina DNA Prep (Illumina, San Diego, CA, USA) and sequenced using Illumina technology (MiSeq). Consensus sequences were made by reference-based mapping towards A/Black_legged_kittiwake/Norway/2023-07-650-2T/2023 (EPI_ISL_18455264) in InsaFlu (*15*).

Sequence analyses

Genetic characterization of the detected viruses was performed, and BLAST (*16*) in GISAID (*17*) was used for similarity searches. Phylogenetic analysis compared the nucleotide sequences of viruses from the ten necropsied Kittiwakes with AIV sequences from Norway (March-July 2023) and selected European sequences (2020-2023) from GISAID EpiFlu. An overview of all sequences included in the analyses is given in the Appendix 2. For each segment, the full-length coding nucleotide sequences were aligned by Clustal W and phylogenetic trees were created using the Maximum likelihood algorithm and the Tamura Nei model in MEGA 10.1.8 (*18*, *19*). We used bootstrap values of 1000 replicates to assess the nodal support. Mutation analyses were performed to search for mutations or motifs previously associated with mammalian viral adaptation or antiviral resistance, using FluMutGUI 3.1.1; FluMut 0.6.3; FluMutDB 6.3, released on 2024-09-12 (https://github.com/izsvenezie-virology/FluMut).

Results

Table S1. Number of dead birds reported by the municipalities in Troms and Finnmark County, Norway, between July 28 and August 28, 2023, due to the HPAI H5N1 outbreak in Kittiwakes (*Rissa tridactyla*) in Northern Norway (*20*). An empty cell means that no data was reported.

Municipality	July 28	August 1	August 4	Week 32	Week 33	Week 34	Total
Alta	140	100		30	0		270
Balsfjord	8	2		3			13
Bardu							0
Berlevåg	358		23	3			384
Båtsfjord		9					9
Dyrøy	0	0	2	5	0	0	7
Gamvik		600		400	500		1500
Gratangen	0	2	0	0	0		2
Hammerfest	2000	229	46	67	16	14	2372
Harstad		5	2	8			15
Hasvik	400	100		330	0		830
Ibestad	6		3	0			9
Karasjok		0					0
Karlsøy	10	13	0	0	0	0	23
Kautokeino	1	0	0				1
Kvæfjord	279	680	97	251	42	20	1369
Kvænangen	25	150	100	0	40		315
Kåfjord	50	5		0			55
Lavangen	0	0	0	0			0
Lebesby	500	40	0	8	0		548
Loppa	100	81		0			181

Lyngen	143	21	0	2	0	0	166
Målselv	4		8	0			12
Måsøy		2					2
Nesseby	40						40
Nordkapp		60					60
Nordreisa	7	0	0	0	13		20
Porsanger	2						2
Salangen	0	5	0	0	0	0	5
Senja	0	0	3	0	0	0	3
Skjervøy	12	6	0	5			23
Storfjord	5	1	2	0	0	0	8
Sørreisa	0	0	0	0			0
Sør-Varanger		75					75
Tana	0	0	0	0	1		1
Tjeldsund	2	4	1	4			11
Tromsø	7	6	2	6	1	6	28
Vadsø	12593	1543	891	208			15235
Vardø	1000						1000
Total	17692	3739	1180	1330	613	40	24594

Table S2. Counts of apparently occupied nests during the outbreak, used for estimation of the number of breeding pairs of Black-legged Kittiwakes at the Ekkerøy colony, Norway, in 2023.

Category	Number of nests
At least one adult and one chick on nest	335
At least one living chick but no adult(s) on nest	669
One or more adults and no chick(s) on nest	4976
Nest used in 2023, but no birds present	1423
Dead chick(s) on nest	414
Uncertainty of occupied nest	111
Total apparently occupied nests (included uncertain nests)	7928
Total apparently occupied nests (without uncertain nests)	7817

Table S3. Individual data and Pectoral Muscle Condition Score (PMCS) of ten Black-legged Kittiwakes that died from HPAI H5N1 and were necropsied at Storelva, Norway, in July 2023.

Bird no.	1	2	3	4	5	6	7	8	9	10
Weight (g)	312	378	310	312	321	308	374	287	266	294
Sex*	F	M	M	F	F	F	M	M	F	F
Head (cm)	9.1	9.6	-	-	9.0	8.8	9.5	9.0	8.4	8.9
PMCS**	3	3	2	1	2	2	1	1	1	2

*F: Female, M: Male

**1: emaciated, 2: moderate condition, 3: good condition

Table S4. Score of histopathological examination: Hematoxylin and eosin (HE)-stained slides of organs from ten Black-legged Kittiwakes that died from HPAI H5N1 at Ekkerøy, Norway, in 2023, were examined and scored semi-quantitatively based on histopathological observations. The severity of changes was noted as: none (0), mild (1), moderate (2) and severe (3). Missing organ was noted as: (-).

Bird number	1	2	3	4	5	6	7	8	9	10
Brain	1	1	2	2	2	2	2	2	2	2
Pancreas	2	2	2	2	2	2	3	2	3	2
Liver	1	2	1	2	1	0	1	1	2	1
Spleen	-	0	0	0	0	0	0	0	0	0
Heart	0	0	1	0	0	0	0	0	0	0
Kidney	0	0	0	0	0	0	0	0	0	0
Lung	0	0	0	0	0	0	0	0	0	0
Proventriculus	0	0	0	0	0	0	0	0	0	-

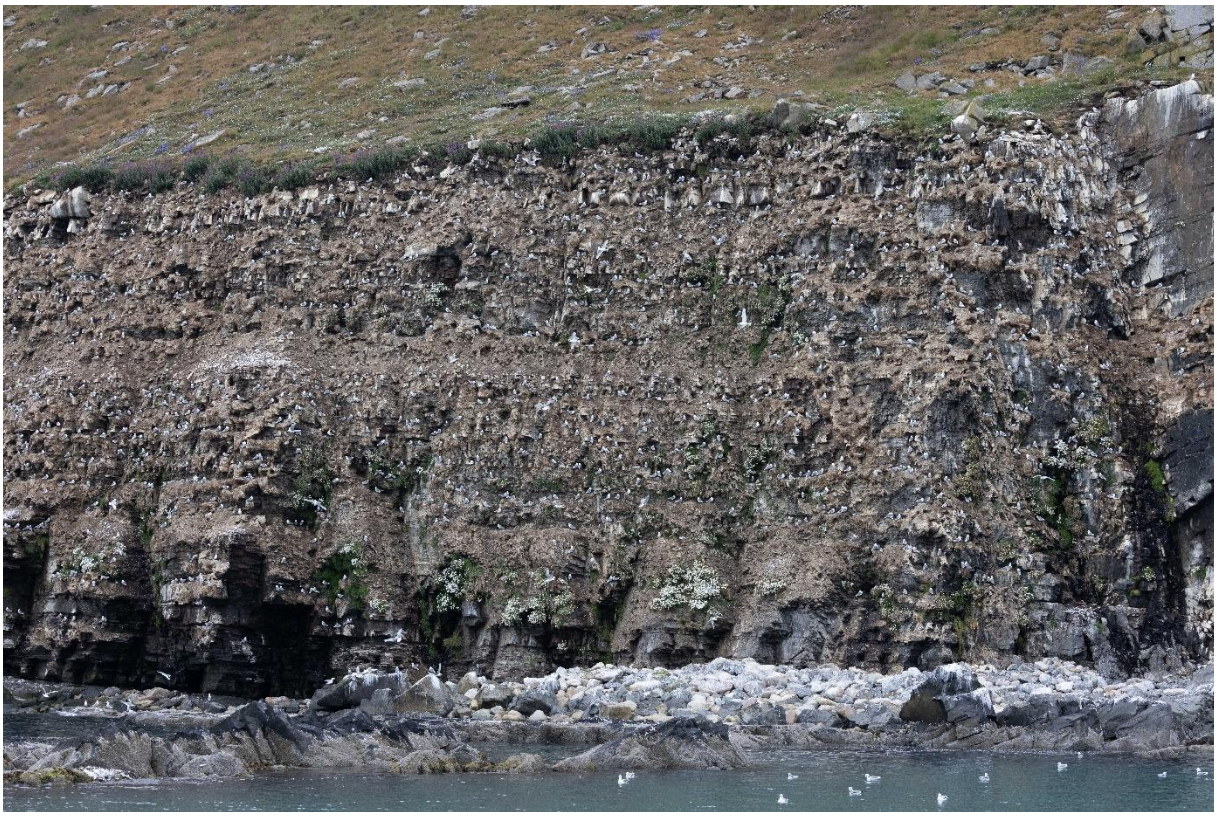

Figure S1. A photo segment of the Kittiwake cliff on Ekkerøy from July 2023, showcasing the image resolution. Apparently occupied nests were counted from several such pictures to estimate the size of the breeding population in 2023. Photo: Knut Sverre Horn.

A)

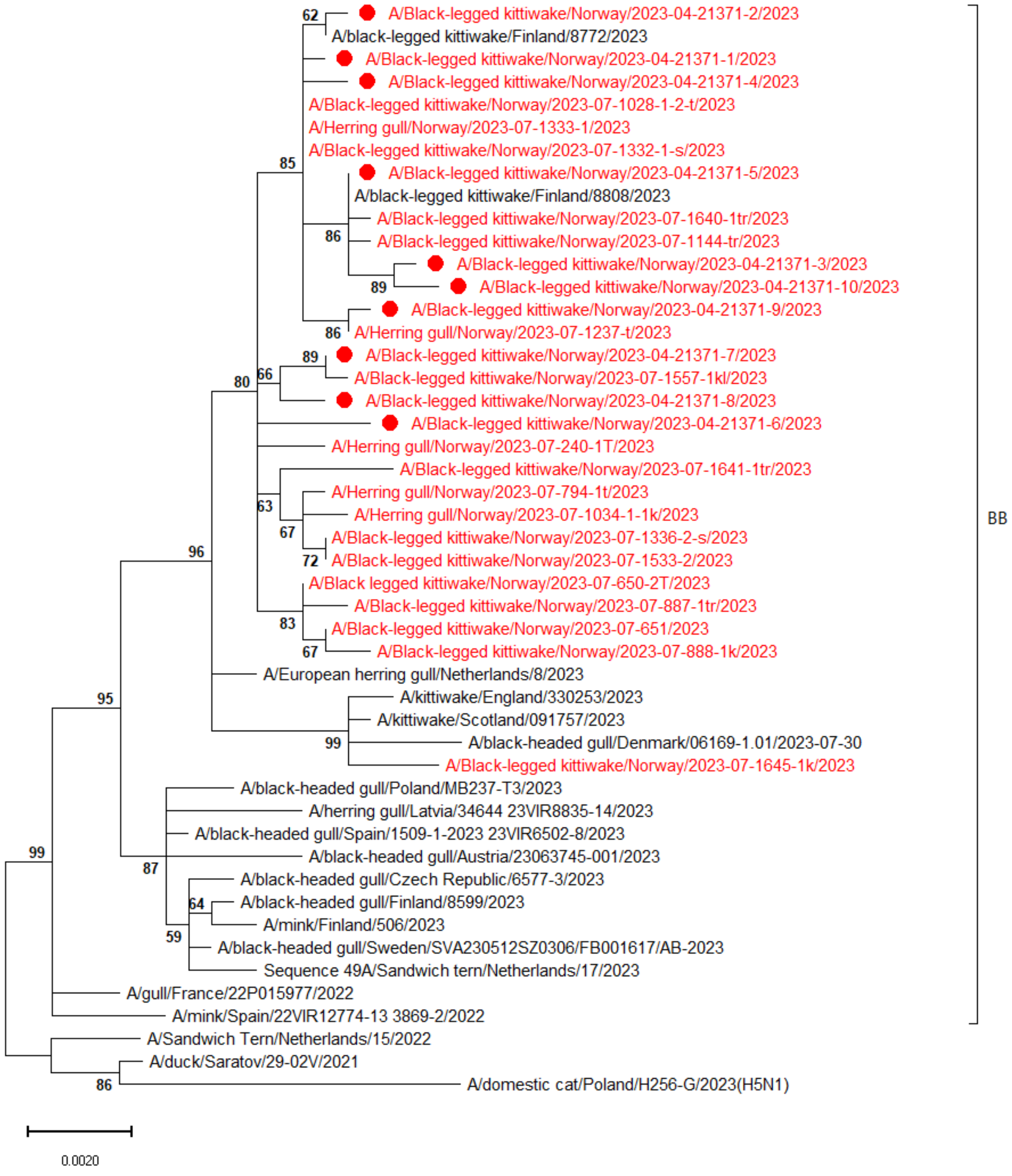

A/Black-legged kittiwake/Norway/2023-04-21371-2/2023
A/black-legged kittiwake/Finland/8772/2023
A/Black-legged kittiwake/Norway/2023-04-21371-1/2023
A/Black-legged kittiwake/Norway/2023-04-21371-4/2023
A/Black-legged kittiwake/Norway/2023-07-1028-1-2-t/2023
A/Herring gull/Norway/2023-07-1333-1/2023
A/Black-legged kittiwake/Norway/2023-07-1332-1-s/2023
A/Black-legged kittiwake/Norway/2023-04-21371-5/2023
A/black-legged kittiwake/Finland/8808/2023
A/Black-legged kittiwake/Norway/2023-07-1640-1tr/2023
A/Black-legged kittiwake/Norway/2023-07-1144-tr/2023
A/Black-legged kittiwake/Norway/2023-04-21371-3/2023
A/Black-legged kittiwake/Norway/2023-04-21371-10/2023
A/Black-legged kittiwake/Norway/2023-04-21371-9/2023
A/Herring gull/Norway/2023-07-1237-t/2023
A/Black-legged kittiwake/Norway/2023-04-21371-7/2023
A/Black-legged kittiwake/Norway/2023-07-1557-1kl/2023
A/Black-legged kittiwake/Norway/2023-04-21371-8/2023
A/Black-legged kittiwake/Norway/2023-04-21371-6/2023
A/Herring gull/Norway/2023-07-240-1T/2023
A/Black-legged kittiwake/Norway/2023-07-1641-1tr/2023
A/Herring gull/Norway/2023-07-794-1t/2023
A/Herring gull/Norway/2023-07-1034-1-1k/2023
A/Black-legged kittiwake/Norway/2023-07-1336-2-s/2023
A/Black-legged kittiwake/Norway/2023-07-1533-2/2023
A/Black legged kittiwake/Norway/2023-07-650-2T/2023
A/Black-legged kittiwake/Norway/2023-07-887-1tr/2023
A/Black-legged kittiwake/Norway/2023-07-651/2023
A/Black-legged kittiwake/Norway/2023-07-888-1k/2023
A/European herring gull/Netherlands/8/2023
A/kittiwake/England/330253/2023
A/kittiwake/Scotland/091757/2023
A/black-headed gull/Denmark/06169-1.01/2023-07-30
A/Black-legged kittiwake/Norway/2023-07-1645-1k/2023
A/black-headed gull/Poland/MB237-T3/2023
A/herring gull/Latvia/34644 23VIR8835-14/2023
A/black-headed gull/Spain/1509-1-2023 23VIR6502-8/2023
A/black-headed gull/Austria/23063745-001/2023
A/black-headed gull/Czech Republic/6577-3/2023
A/black-headed gull/Finland/8599/2023
A/mink/Finland/506/2023
A/black-headed gull/Sweden/SVA230512SZ0306/FB001617/AB-2023
Sequence 49A/Sandwich tern/Netherlands/17/2023
A/gull/France/22P015977/2022
A/mink/Spain/22VIR12774-13 3869-2/2022
A/Sandwich Tern/Netherlands/15/2022
A/duck/Saratov/29-02V/2021
A/domestic cat/Poland/H256-G/2023(H5N1)
62
85
86
89
86
89
66
80
63
67
72
83
67
96
99
95
87
64
59
99
86
BB
0.0020

B)

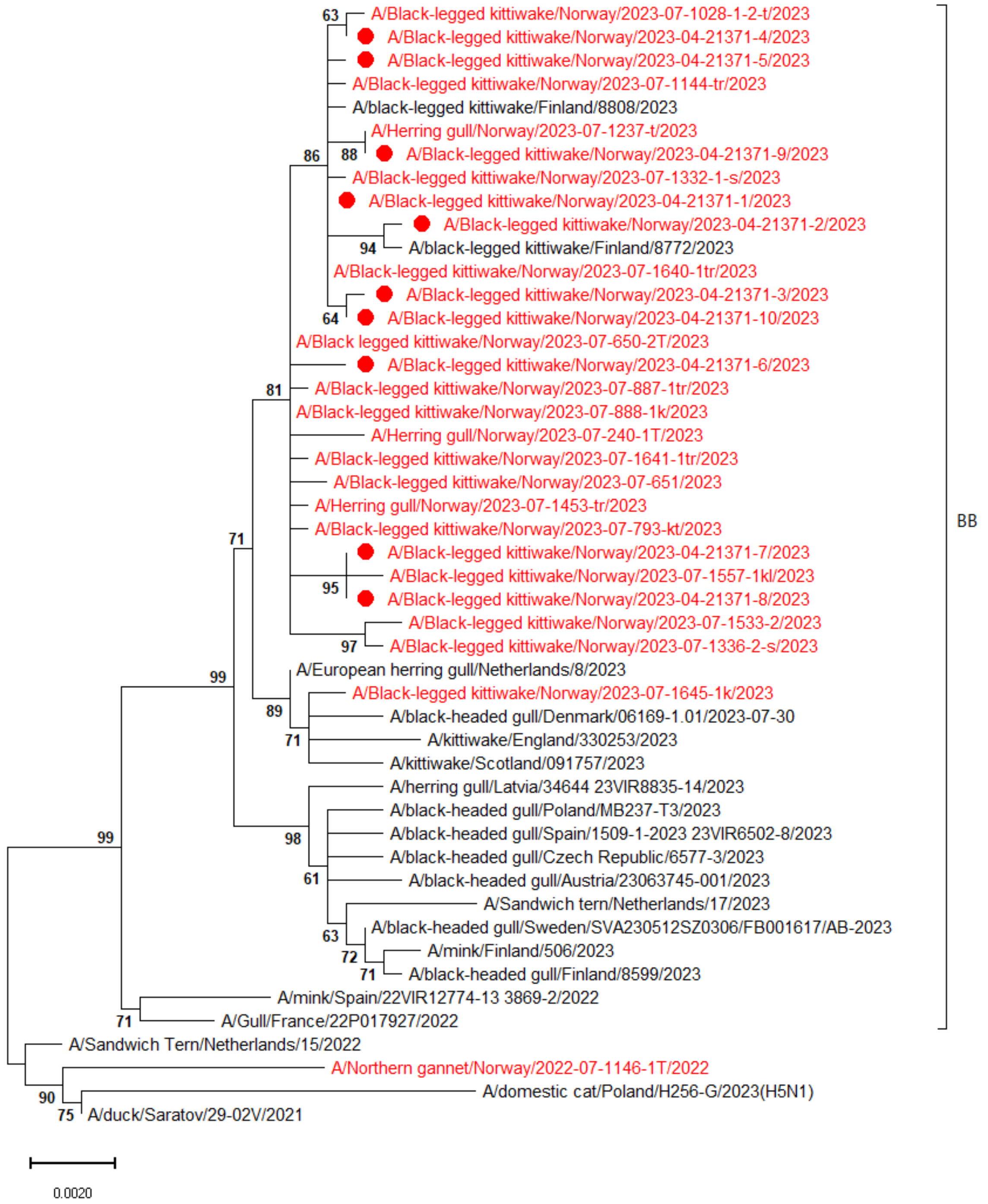
A/Black-legged kittiwake/Norway/2023-07-1028-1-2-t/2023
A/Black-legged kittiwake/Norway/2023-04-21371-4/2023
A/Black-legged kittiwake/Norway/2023-04-21371-5/2023
A/Black-legged kittiwake/Norway/2023-07-1144-tr/2023
A/black-legged kittiwake/Finland/8808/2023
A/Herring gull/Norway/2023-07-1237-t/2023
A/Black-legged kittiwake/Norway/2023-04-21371-9/2023
A/Black-legged kittiwake/Norway/2023-07-1332-1-s/2023
A/Black-legged kittiwake/Norway/2023-04-21371-1/2023
A/Black-legged kittiwake/Norway/2023-04-21371-2/2023
A/black-legged kittiwake/Finland/8772/2023
A/Black-legged kittiwake/Norway/2023-07-1640-1tr/2023
A/Black-legged kittiwake/Norway/2023-04-21371-3/2023
A/Black-legged kittiwake/Norway/2023-04-21371-10/2023
A/Black legged kittiwake/Norway/2023-07-650-2T/2023
A/Black-legged kittiwake/Norway/2023-04-21371-6/2023
A/Black-legged kittiwake/Norway/2023-07-887-1tr/2023
A/Black-legged kittiwake/Norway/2023-07-888-1k/2023
A/Herring gull/Norway/2023-07-240-1T/2023
A/Black-legged kittiwake/Norway/2023-07-1641-1tr/2023
A/Black-legged kittiwake/Norway/2023-07-651/2023
A/Herring gull/Norway/2023-07-1453-tr/2023
A/Black-legged kittiwake/Norway/2023-07-793-kt/2023
A/Black-legged kittiwake/Norway/2023-04-21371-7/2023
A/Black-legged kittiwake/Norway/2023-07-1557-1kl/2023
A/Black-legged kittiwake/Norway/2023-04-21371-8/2023
A/Black-legged kittiwake/Norway/2023-07-1533-2/2023
A/Black-legged kittiwake/Norway/2023-07-1336-2-s/2023
A/European herring gull/Netherlands/8/2023
A/Black-legged kittiwake/Norway/2023-07-1645-1k/2023
A/black-headed gull/Denmark/06169-1.01/2023-07-30
A/kittiwake/England/330253/2023
A/kittiwake/Scotland/091757/2023
A/herring gull/Latvia/34644 23VIR8835-14/2023
A/black-headed gull/Poland/MB237-T3/2023
A/black-headed gull/Spain/1509-1-2023 23VIR6502-8/2023
A/black-headed gull/Czech Republic/6577-3/2023
A/black-headed gull/Austria/23063745-001/2023
A/Sandwich tern/Netherlands/17/2023
A/black-headed gull/Sweden/SVA230512SZ0306/FB001617/AB-2023
A/mink/Finland/506/2023
A/black-headed gull/Finland/8599/2023
A/mink/Spain/22VIR12774-13 3869-2/2022
A/Gull/France/22P017927/2022
A/Sandwich Tern/Netherlands/15/2022
A/Northern gannet/Norway/2022-07-1146-1T/2022
A/domestic cat/Poland/H256-G/2023(H5N1)
A/duck/Saratov/29-02V/2021
63
86
88
94
64
81
95
97
71
89
71
99
98
61
63
72
71
99
71
90
75
BB
0.0020

C)

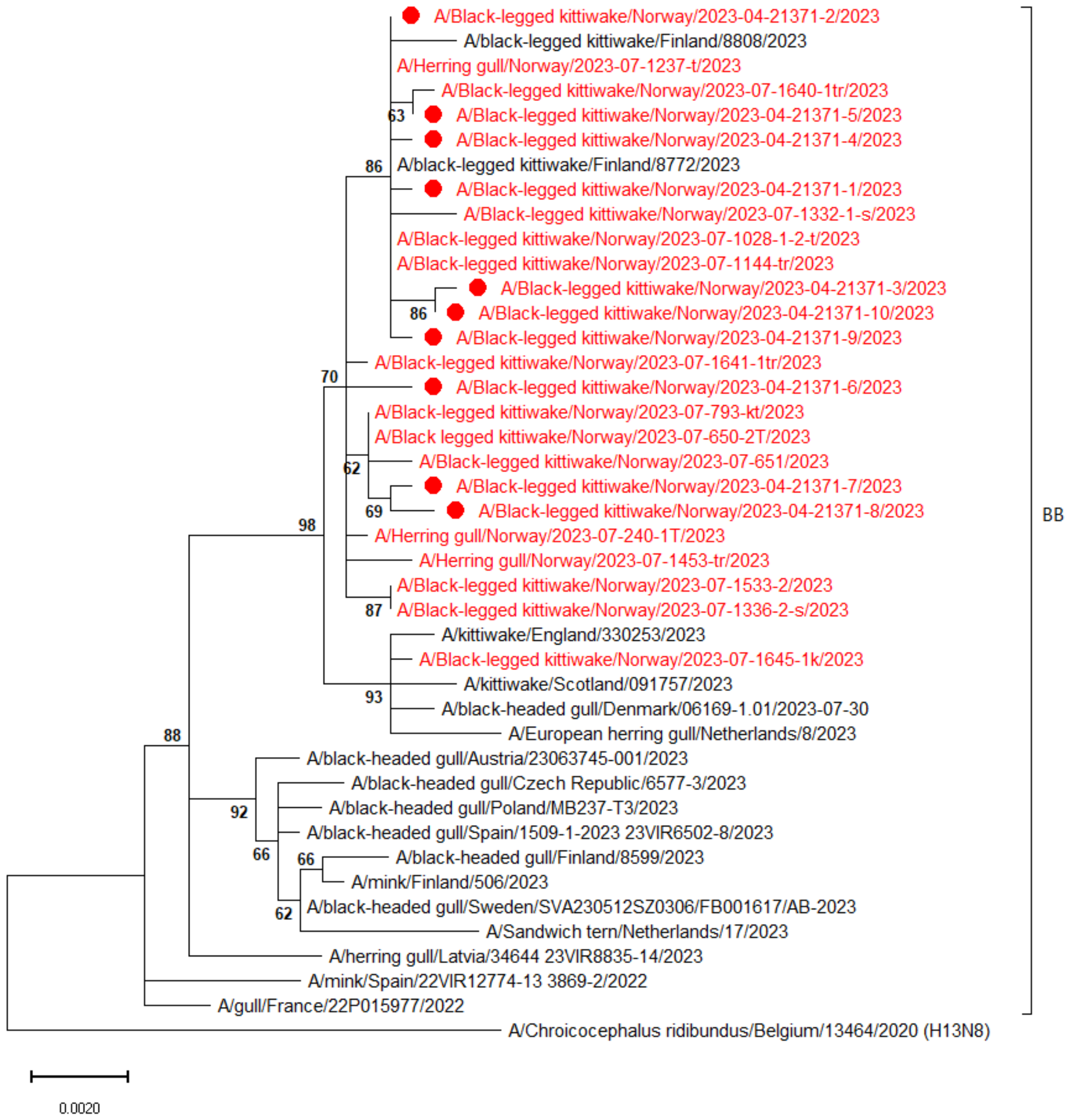
A/Black-legged kittiwake/Norway/2023-04-21371-2/2023
A/black-legged kittiwake/Finland/8808/2023
A/Herring gull/Norway/2023-07-1237-t/2023
A/Black-legged kittiwake/Norway/2023-07-1640-1tr/2023
A/Black-legged kittiwake/Norway/2023-04-21371-5/2023
A/Black-legged kittiwake/Norway/2023-04-21371-4/2023
A/black-legged kittiwake/Finland/8772/2023
A/Black-legged kittiwake/Norway/2023-04-21371-1/2023
A/Black-legged kittiwake/Norway/2023-07-1332-1-s/2023
A/Black-legged kittiwake/Norway/2023-07-1028-1-2-t/2023
A/Black-legged kittiwake/Norway/2023-07-1144-tr/2023
A/Black-legged kittiwake/Norway/2023-04-21371-3/2023
A/Black-legged kittiwake/Norway/2023-04-21371-10/2023
A/Black-legged kittiwake/Norway/2023-04-21371-9/2023
A/Black-legged kittiwake/Norway/2023-07-1641-1tr/2023
A/Black-legged kittiwake/Norway/2023-04-21371-6/2023
A/Black-legged kittiwake/Norway/2023-07-793-kt/2023
A/Black legged kittiwake/Norway/2023-07-650-2T/2023
A/Black-legged kittiwake/Norway/2023-07-651/2023
A/Black-legged kittiwake/Norway/2023-04-21371-7/2023
A/Black-legged kittiwake/Norway/2023-04-21371-8/2023
A/Herring gull/Norway/2023-07-240-1T/2023
A/Herring gull/Norway/2023-07-1453-tr/2023
A/Black-legged kittiwake/Norway/2023-07-1533-2/2023
A/Black-legged kittiwake/Norway/2023-07-1336-2-s/2023
A/kittiwake/England/330253/2023
A/Black-legged kittiwake/Norway/2023-07-1645-1k/2023
A/kittiwake/Scotland/091757/2023
A/black-headed gull/Denmark/06169-1.01/2023-07-30
A/European herring gull/Netherlands/8/2023
A/black-headed gull/Austria/23063745-001/2023
A/black-headed gull/Czech Republic/6577-3/2023
A/black-headed gull/Poland/MB237-T3/2023
A/black-headed gull/Spain/1509-1-2023 23VIR6502-8/2023
A/black-headed gull/Finland/8599/2023
A/mink/Finland/506/2023
A/black-headed gull/Sweden/SVA230512SZ0306/FB001617/AB-2023
A/Sandwich tern/Netherlands/17/2023
A/herring gull/Latvia/34644 23VIR8835-14/2023
A/mink/Spain/22VIR12774-13 3869-2/2022
A/gull/France/22P015977/2022
A/Chroicocephalus ridibundus/Belgium/13464/2020 (H13N8)
63
86
86
70
62
69
98
87
93
88
92
66
66
62
BB
0.0020

D)

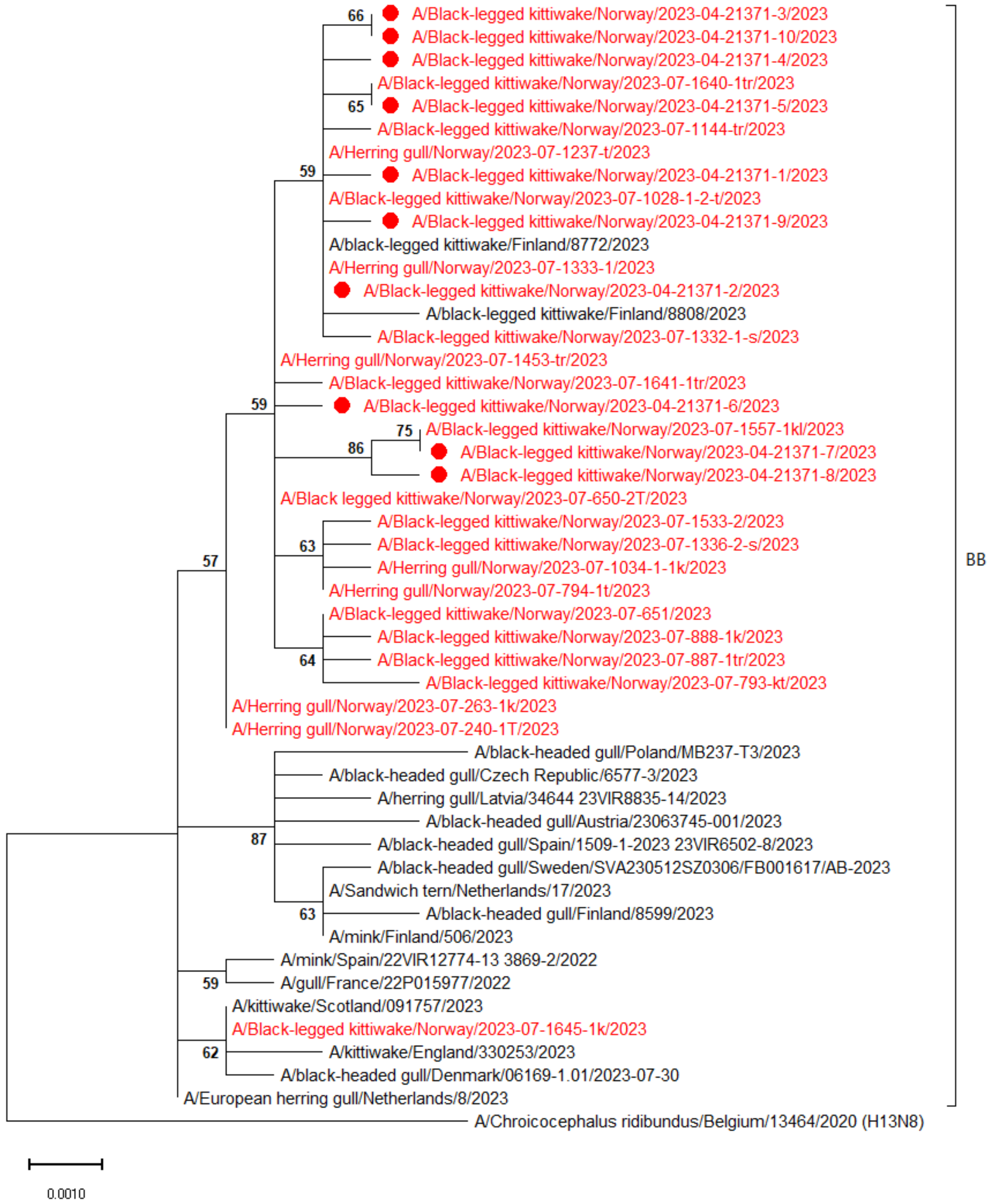

66
A/Black-legged kittiwake/Norway/2023-04-21371-3/2023
A/Black-legged kittiwake/Norway/2023-04-21371-10/2023
A/Black-legged kittiwake/Norway/2023-04-21371-4/2023
A/Black-legged kittiwake/Norway/2023-07-1640-1tr/2023
65
A/Black-legged kittiwake/Norway/2023-04-21371-5/2023
A/Black-legged kittiwake/Norway/2023-07-1144-tr/2023
A/Herring gull/Norway/2023-07-1237-t/2023
59
A/Black-legged kittiwake/Norway/2023-04-21371-1/2023
A/Black-legged kittiwake/Norway/2023-07-1028-1-2-t/2023
A/Black-legged kittiwake/Norway/2023-04-21371-9/2023
A/black-legged kittiwake/Finland/8772/2023
A/Herring gull/Norway/2023-07-1333-1/2023
A/Black-legged kittiwake/Norway/2023-04-21371-2/2023
A/black-legged kittiwake/Finland/8808/2023
A/Black-legged kittiwake/Norway/2023-07-1332-1-s/2023
A/Herring gull/Norway/2023-07-1453-tr/2023
A/Black-legged kittiwake/Norway/2023-07-1641-1tr/2023
59
A/Black-legged kittiwake/Norway/2023-04-21371-6/2023
75
A/Black-legged kittiwake/Norway/2023-07-1557-1kl/2023
86
A/Black-legged kittiwake/Norway/2023-04-21371-7/2023
A/Black-legged kittiwake/Norway/2023-04-21371-8/2023
A/Black legged kittiwake/Norway/2023-07-650-2T/2023
A/Black-legged kittiwake/Norway/2023-07-1533-2/2023
63
A/Black-legged kittiwake/Norway/2023-07-1336-2-s/2023
57
A/Herring gull/Norway/2023-07-1034-1-1k/2023
A/Herring gull/Norway/2023-07-794-1t/2023
A/Black-legged kittiwake/Norway/2023-07-651/2023
A/Black-legged kittiwake/Norway/2023-07-888-1k/2023
64
A/Black-legged kittiwake/Norway/2023-07-887-1tr/2023
A/Black-legged kittiwake/Norway/2023-07-793-kt/2023
A/Herring gull/Norway/2023-07-263-1k/2023
A/Herring gull/Norway/2023-07-240-1T/2023
A/black-headed gull/Poland/MB237-T3/2023
A/black-headed gull/Czech Republic/6577-3/2023
A/herring gull/Latvia/34644 23VIR8835-14/2023
A/black-headed gull/Austria/23063745-001/2023
87
A/black-headed gull/Spain/1509-1-2023 23VIR6502-8/2023
A/black-headed gull/Sweden/SVA230512SZ0306/FB001617/AB-2023
A/Sandwich tern/Netherlands/17/2023
63
A/black-headed gull/Finland/8599/2023
A/mink/Finland/506/2023
A/mink/Spain/22VIR12774-13 3869-2/2022
59
A/gull/France/22P015977/2022
A/kittiwake/Scotland/091757/2023
A/Black-legged kittiwake/Norway/2023-07-1645-1k/2023
62
A/kittiwake/England/330253/2023
A/black-headed gull/Denmark/06169-1.01/2023-07-30
A/European herring gull/Netherlands/8/2023
A/Chroicocephalus ridibundus/Belgium/13464/2020 (H13N8)
BB
0.0010

E)

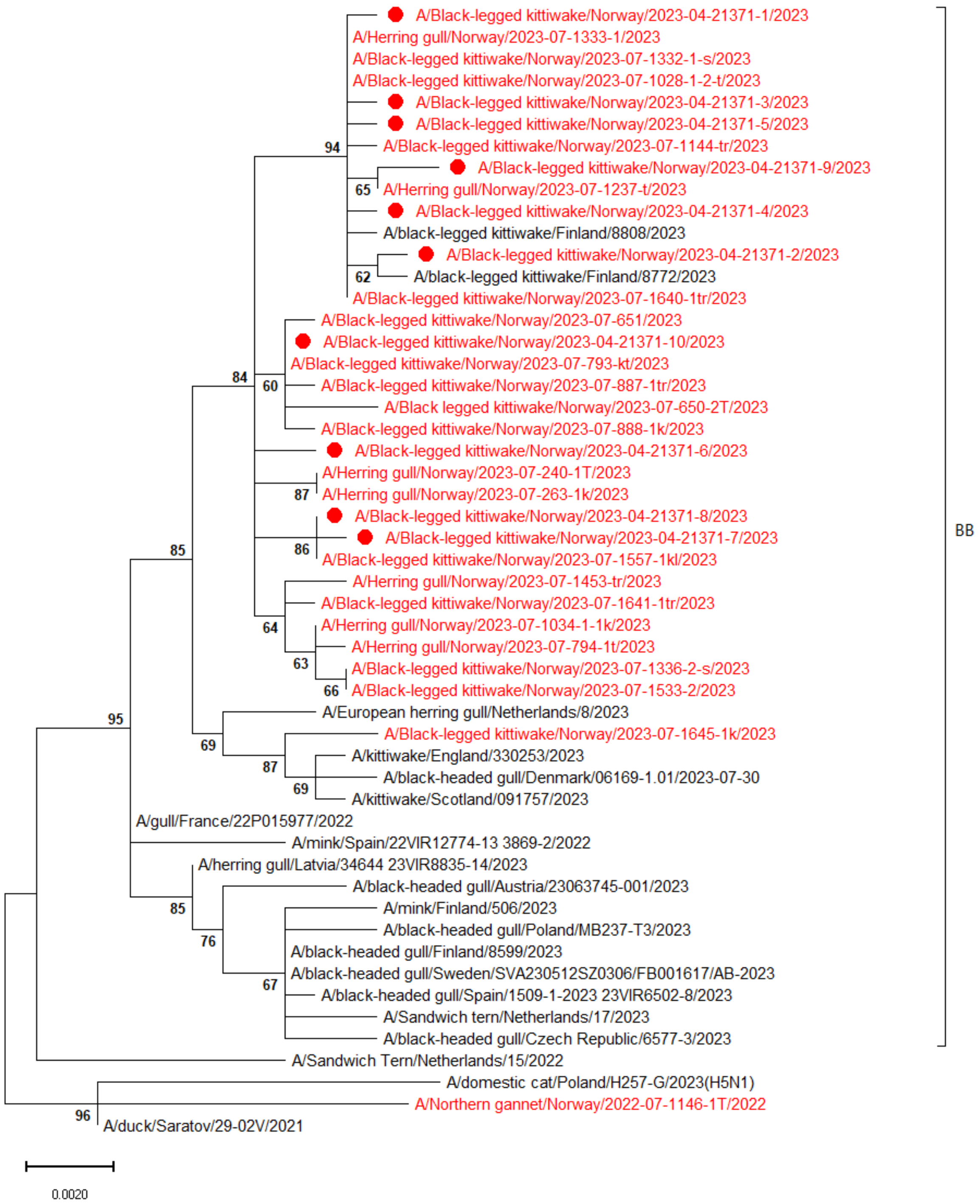
A/Black-legged kittiwake/Norway/2023-04-21371-1/2023
A/Herring gull/Norway/2023-07-1333-1/2023
A/Black-legged kittiwake/Norway/2023-07-1332-1-s/2023
A/Black-legged kittiwake/Norway/2023-07-1028-1-2-t/2023
A/Black-legged kittiwake/Norway/2023-04-21371-3/2023
A/Black-legged kittiwake/Norway/2023-04-21371-5/2023
94
A/Black-legged kittiwake/Norway/2023-07-1144-tr/2023
A/Black-legged kittiwake/Norway/2023-04-21371-9/2023
65
A/Herring gull/Norway/2023-07-1237-t/2023
A/Black-legged kittiwake/Norway/2023-04-21371-4/2023
A/black-legged kittiwake/Finland/8808/2023
A/Black-legged kittiwake/Norway/2023-04-21371-2/2023
62
A/black-legged kittiwake/Finland/8772/2023
A/Black-legged kittiwake/Norway/2023-07-1640-1tr/2023
A/Black-legged kittiwake/Norway/2023-07-651/2023
A/Black-legged kittiwake/Norway/2023-04-21371-10/2023
A/Black-legged kittiwake/Norway/2023-07-793-kt/2023
84
60
A/Black-legged kittiwake/Norway/2023-07-887-1tr/2023
A/Black legged kittiwake/Norway/2023-07-650-2T/2023
A/Black-legged kittiwake/Norway/2023-07-888-1k/2023
A/Black-legged kittiwake/Norway/2023-04-21371-6/2023
A/Herring gull/Norway/2023-07-240-1T/2023
87
A/Herring gull/Norway/2023-07-263-1k/2023
A/Black-legged kittiwake/Norway/2023-04-21371-8/2023
85
86
A/Black-legged kittiwake/Norway/2023-04-21371-7/2023
A/Black-legged kittiwake/Norway/2023-07-1557-1kl/2023
A/Herring gull/Norway/2023-07-1453-tr/2023
A/Black-legged kittiwake/Norway/2023-07-1641-1tr/2023
64
A/Herring gull/Norway/2023-07-1034-1-1k/2023
A/Herring gull/Norway/2023-07-794-1t/2023
63
A/Black-legged kittiwake/Norway/2023-07-1336-2-s/2023
66
A/Black-legged kittiwake/Norway/2023-07-1533-2/2023
A/European herring gull/Netherlands/8/2023
95
A/Black-legged kittiwake/Norway/2023-07-1645-1k/2023
69
A/kittiwake/England/330253/2023
87
A/black-headed gull/Denmark/06169-1.01/2023-07-30
69
A/kittiwake/Scotland/091757/2023
A/gull/France/22P015977/2022
A/mink/Spain/22VIR12774-13 3869-2/2022
A/herring gull/Latvia/34644 23VIR8835-14/2023
A/black-headed gull/Austria/23063745-001/2023
85
A/mink/Finland/506/2023
76
A/black-headed gull/Poland/MB237-T3/2023
A/black-headed gull/Finland/8599/2023
A/black-headed gull/Sweden/SVA230512SZ0306/FB001617/AB-2023
67
A/black-headed gull/Spain/1509-1-2023 23VIR6502-8/2023
A/Sandwich tern/Netherlands/17/2023
A/black-headed gull/Czech Republic/6577-3/2023
A/Sandwich Tern/Netherlands/15/2022
A/domestic cat/Poland/H257-G/2023(H5N1)
A/Northern gannet/Norway/2022-07-1146-1T/2022
96
A/duck/Saratov/29-02V/2021
BB
0.0020

F)

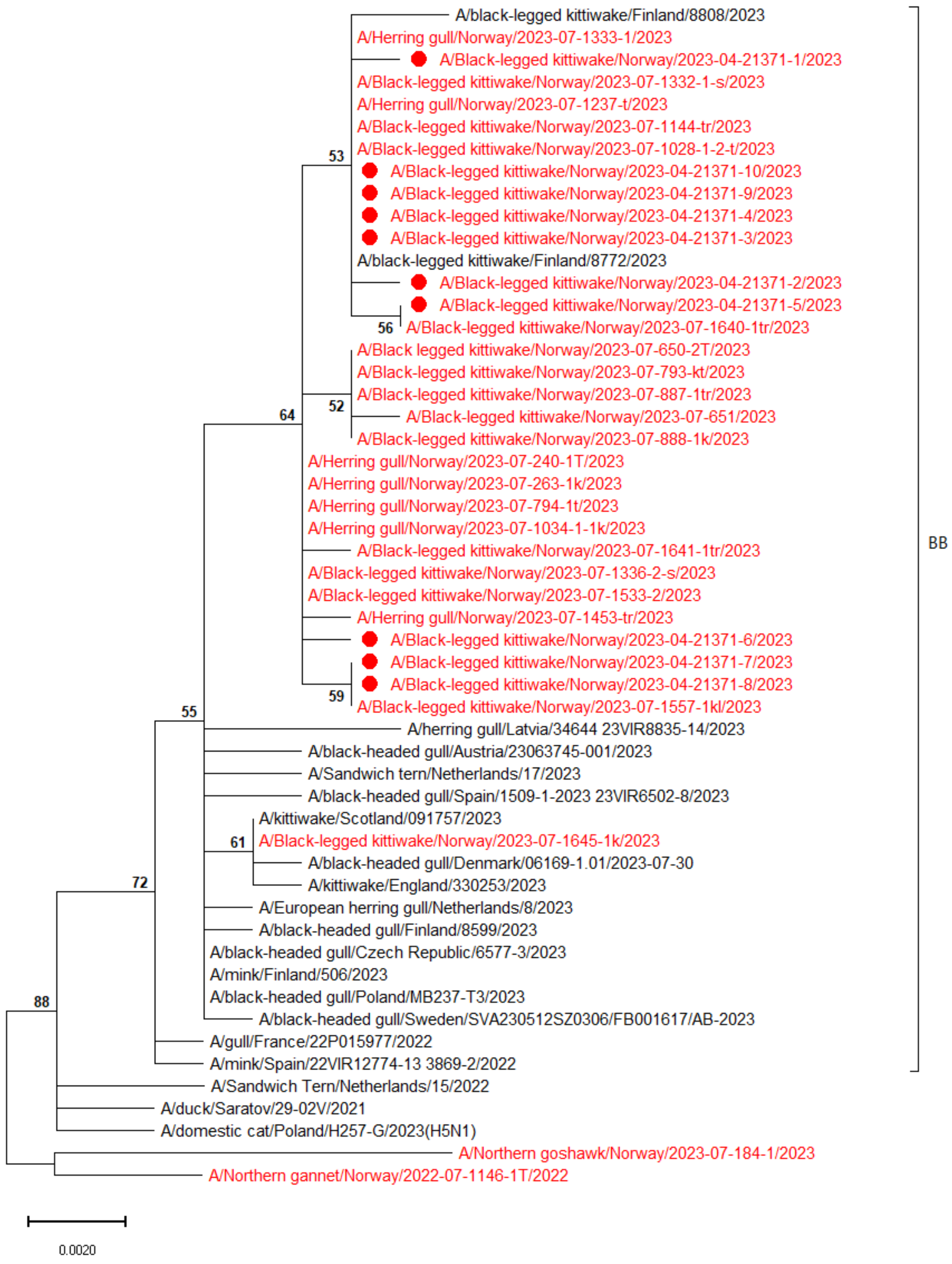
A/black-legged kittiwake/Finland/8808/2023
A/Herring gull/Norway/2023-07-1333-1/2023
A/Black-legged kittiwake/Norway/2023-04-21371-1/2023
A/Black-legged kittiwake/Norway/2023-07-1332-1-s/2023
A/Herring gull/Norway/2023-07-1237-t/2023
A/Black-legged kittiwake/Norway/2023-07-1144-tr/2023
53
A/Black-legged kittiwake/Norway/2023-07-1028-1-2-t/2023
A/Black-legged kittiwake/Norway/2023-04-21371-10/2023
A/Black-legged kittiwake/Norway/2023-04-21371-9/2023
A/Black-legged kittiwake/Norway/2023-04-21371-4/2023
A/Black-legged kittiwake/Norway/2023-04-21371-3/2023
A/black-legged kittiwake/Finland/8772/2023
A/Black-legged kittiwake/Norway/2023-04-21371-2/2023
A/Black-legged kittiwake/Norway/2023-04-21371-5/2023
56
A/Black-legged kittiwake/Norway/2023-07-1640-1tr/2023
A/Black legged kittiwake/Norway/2023-07-650-2T/2023
A/Black-legged kittiwake/Norway/2023-07-793-kt/2023
A/Black-legged kittiwake/Norway/2023-07-887-1tr/2023
52
A/Black-legged kittiwake/Norway/2023-07-651/2023
64
A/Black-legged kittiwake/Norway/2023-07-888-1k/2023
A/Herring gull/Norway/2023-07-240-1T/2023
A/Herring gull/Norway/2023-07-263-1k/2023
A/Herring gull/Norway/2023-07-794-1t/2023
A/Herring gull/Norway/2023-07-1034-1-1k/2023
A/Black-legged kittiwake/Norway/2023-07-1641-1tr/2023
A/Black-legged kittiwake/Norway/2023-07-1336-2-s/2023
A/Black-legged kittiwake/Norway/2023-07-1533-2/2023
A/Herring gull/Norway/2023-07-1453-tr/2023
A/Black-legged kittiwake/Norway/2023-04-21371-6/2023
A/Black-legged kittiwake/Norway/2023-04-21371-7/2023
A/Black-legged kittiwake/Norway/2023-04-21371-8/2023
59
A/Black-legged kittiwake/Norway/2023-07-1557-1kl/2023
55
A/herring gull/Latvia/34644 23VIR8835-14/2023
A/black-headed gull/Austria/23063745-001/2023
A/Sandwich tern/Netherlands/17/2023
A/black-headed gull/Spain/1509-1-2023 23VIR6502-8/2023
A/kittiwake/Scotland/091757/2023
61
A/Black-legged kittiwake/Norway/2023-07-1645-1k/2023
A/black-headed gull/Denmark/06169-1.01/2023-07-30
A/kittiwake/England/330253/2023
72
A/European herring gull/Netherlands/8/2023
A/black-headed gull/Finland/8599/2023
A/black-headed gull/Czech Republic/6577-3/2023
A/mink/Finland/506/2023
88
A/black-headed gull/Poland/MB237-T3/2023
A/black-headed gull/Sweden/SVA230512SZ0306/FB001617/AB-2023
A/gull/France/22P015977/2022
A/mink/Spain/22VIR12774-13 3869-2/2022
A/Sandwich Tern/Netherlands/15/2022
A/duck/Saratov/29-02V/2021
A/domestic cat/Poland/H257-G/2023(H5N1)
A/Northern goshawk/Norway/2023-07-184-1/2023
A/Northern gannet/Norway/2022-07-1146-1T/2022
BB
0.0020

G)

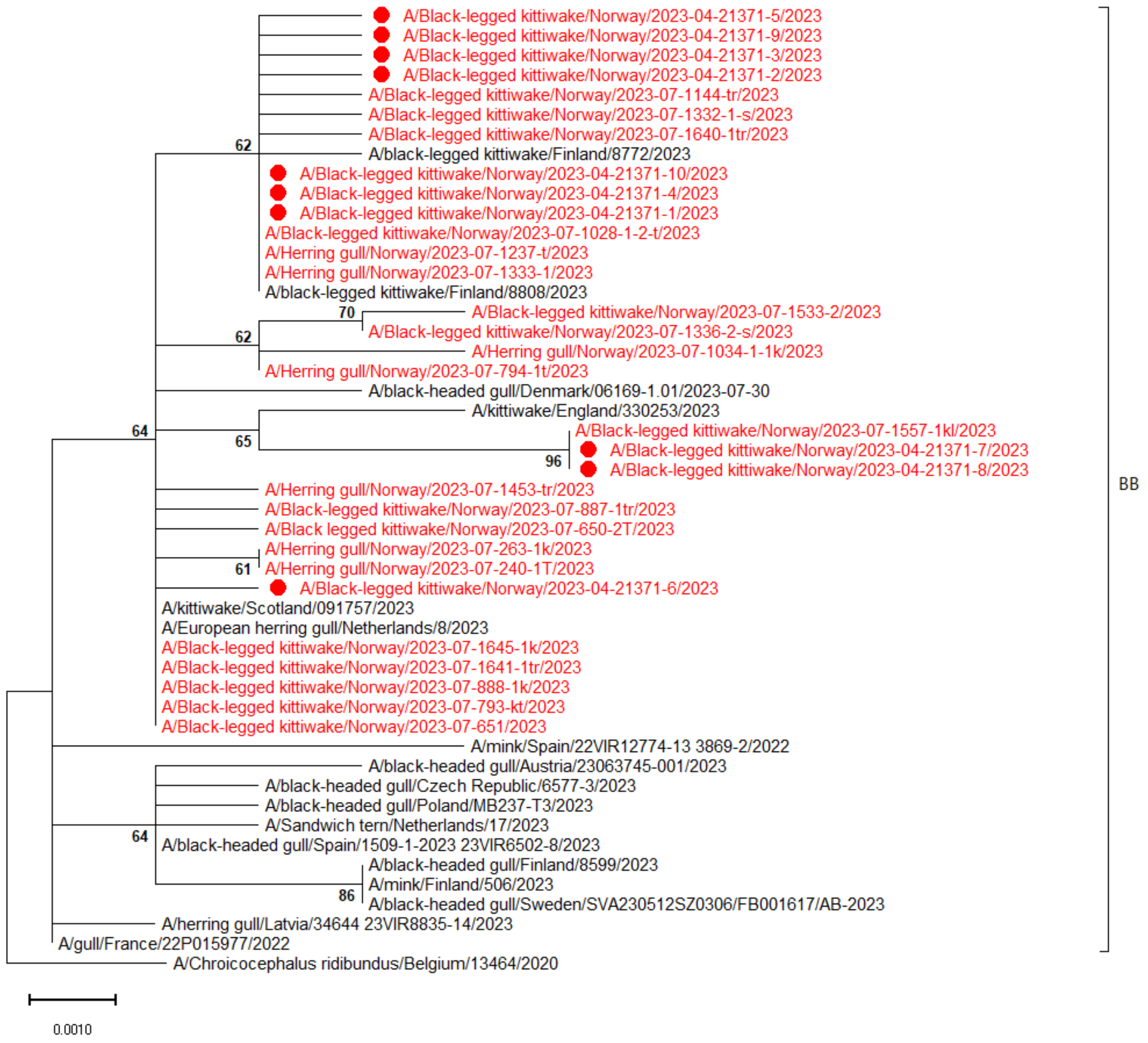

Figure S2. Midpoint rooted phylogenetic trees showing the genetic relationship between ten HPAI H5N1 viruses identified in dead Black-legged Kittiwakes from Ekkerøy, Norway, in 2023 (red dots), and contemporary viruses from Norway (red) and Europe (black), including the coding part of the gene segments A) PB2, B) PB1, C) PA, D) NP, E) NA, F) M, G) NS. Bootstrap values >50 are shown.

References

1. R Core Team. R: A language and environment for statistical computing. Vienna, Austria: R Foundation for Statistical Computing; 2021.
2. Artsdatabanken. The Norwegian Ministry of Climate and Environment.
3. Franeker JAv, Camphuysen CJ. Condition manual: the physical condition of stranded seabirds. Handbook on oil impact assessment; 2007.
4. Gaide N, Crispo M, Jbenyeni A, Bleuart C, Delverdier M, Vergne T, et al. Validation of an RNAscope assay for the detection of avian influenza A virus. J Vet Diagn Invest. 2023 Sep;35(5):500-6.
5. van Riel D, Leijten LM, Kochs G, Osterhaus A, Kuiken T. Decrease of virus receptors during highly pathogenic H5N1 virus infection in humans and other mammals. Am J Pathol. 2013 Nov;183(5):1382-9.
6. Gambaryan A, Yamnikova S, Lvov D, Tuzikov A, Chinarev A, Pazynina G, et al. Receptor specificity of influenza viruses from birds and mammals: new data on involvement of the inner fragments of the carbohydrate chain. Virology. 2005 Apr 10;334(2):276-83.
7. Srivastava S, Verhagen A, Sasmal A, Wasik BR, Diaz S, Yu H, et al. Development and applications of sialoglycan-recognizing probes (SGRPs) with defined specificities: exploring the dynamic mammalian sialoglycome. Glycobiology. 2022 Nov 22;32(12):1116-36.
8. Spackman E, Senne DA, Myers TJ, Bulaga LL, Garber LP, Perdue ML, et al. Development of a real-time reverse transcriptase PCR assay for type A influenza virus and the avian H5 and H7 hemagglutinin subtypes. J Clin Microbiol. 2002 Sep;40(9):3256-60.
9. World Organisation for Animal Health. Avian Influenza (including infection with high pathogenicity avian influenza viruses): Office international des épizooties; 2021.
10. James J, Seekings AH, Skinner P, Purchase K, Mahmood S, Brown IH, et al. Rapid and sensitive detection of high pathogenicity Eurasian clade 2.3.4.4b avian influenza viruses in wild birds and poultry. J Virol Methods. 2022 Mar;301:114454.
11. Naguib MM, Graaf A, Fortin A, Luttermann C, Wernery U, Amarin N, et al. Novel real-time PCR-based patho- and phylotyping of potentially zoonotic avian influenza A subtype H5 viruses at risk of incursion into Europe in 2017. Euro Surveill. 2017 Jan 5;22(1).
12. Payungporn S, Chutinimitkul S, Chaisingh A, Damrongwantanapokin S, Buranathai C, Amonsin A, et al. Single step multiplex real-time RT-PCR for H5N1 influenza A virus detection. J Virol Methods. 2006 Feb;131(2):143-7.
13. Slomka MJ, Pavlidis T, Banks J, Shell W, McNally A, Essen S, et al. Validated H5 Eurasian real-time reverse transcriptase-polymerase chain reaction and its application in H5N1 outbreaks in 2005-2006. Avian Dis. 2007 Mar;51(1 Suppl):373-7.
14. Zhou B, Donnelly ME, Scholes DT, St George K, Hatta M, Kawaoka Y, et al. Single-reaction genomic amplification accelerates sequencing and vaccine production for classical and Swine origin human influenza a viruses. J Virol. 2009 Oct;83(19):10309-13.
15. Borges V, Pinheiro M, Pechirra P, Guiomar R, Gomes JP. INSaFLU: an automated open web-based bioinformatics suite “from-reads” for influenza whole-genome-sequencing-based surveillance. Genome Med. 2018 2018/06/29;10(1):46.
16. Altschul SF, Gish W, Miller W, Myers EW, Lipman DJ. Basic local alignment search tool. J Mol Biol. 1990 1990/10/05/;215(3):403-10.
17. Shu Y, McCauley J. GISAID: Global initiative on sharing all influenza data – from vision to reality. Euro Surveill. 2017;22(13):30494.
18. Kumar S, Stecher G, Li M, Knyaz C, Tamura K. MEGA X: Molecular Evolutionary Genetics Analysis across Computing Platforms. Mol Biol Evol. 2018 Jun 1;35(6):1547-9.

19. Tamura K, Nei M. Estimation of the number of nucleotide substitutions in the control region of mitochondrial DNA in humans and chimpanzees. Mol Biol Evol. 1993 May;10(3):512-26.
20. Finnmark CGoTa. 24.000 dead birds reported. 2023 [cited 2023-08-28]; Available from: https://www.statsforvalteren.no/nb/troms-finnmark/nyheter/2023/08/25.000-dode-fugler-rapportert/
