# Supplementary material for: Highly Pathogenic Avian Influenza A (H5N1) Caused Mass Death Among Black‐Legged Kittiwakes (Rissa tridactyla) in Norway, 2023
Source: Transbound Emerg Dis. 2026 Feb 18;2026:2963364. doi: 10.1155/tbed/2963364 (PMC12917261; doi:10.1155/tbed/2963364)
Supplement: Supplementary file 3 — Supporting Information 3 Appendix 2: Data included in the phylogenetic analyses. [file TBED-2026-2963364-s003.pdf]

Appendix 2 Table 1. Data included in the phylogenetic analyses.

We thank the authors, originating and submitting laboratories of the sequences from GISAID's EpiFlu Database (www.gisaid.org) on which this research is based. A detailed list is found below.

GISAID isolat acc.no	Isolate name (ID, label)	Pathogenicy	Subtype	Clade	Genotype	PB2	PB1	PA	HA	NP	NA	M	NS	Collection date
EPI ISL 18292959	A/black-legged kittiwake/Finland/8808/2023	HPAI	H5N1	2.3.4.4b	BB	EPI2755467	EPI2755468	EPI2755466	EPI2755470	EPI2755463	EPI2755469	EPI2755465	EPI2755464	2023-07-28
EPI ISL 18728075	A/black-legged kittiwake/Finland/8772/2023	HPAI	H5N1	2.3.4.4b	BB	EPI2892377	EPI2892379	EPI2892381	EPI2892391	EPI2892421	EPI2892446	EPI2892471	EPI2892493	2023-07-21
EPI ISL 18755127	A/black-headed gull/Finland/8599/2023	HPAI	H5N1	2.3.4.4b	BB	EPI2907863	EPI2907864	EPI2907865	EPI2907866	EPI2907867	EPI2907868	EPI2907869	EPI2907870	2023-07-20
EPI ISL 18271510	A/black-headed gull/Denmark/06169-1.01/2023-07-3	HPAI	H5N1	2.3.4.4b	BB	EPI2745873	EPI2745874	EPI2745875	EPI2745876	EPI2745877	EPI2745878	EPI2745879	EPI2745880	2023-07-30
EPI ISL 13778522	A/Sandwich Tern/Netherlands/15/2022	HPAI	H5N2	2.3.4.4b	AB	EPI2088979	EPI2088980	Excluded*	EPI2088982	Excluded*	EPI2088981	EPI2088977	Excluded*	2022-06-24
EPI ISL 18882069	A/Sandwich tern/Netherlands/17/2023	HPAI	H5N3	2.3.4.4b	BB	EPI3010420	EPI3010421	EPI3010419	EPI3010423	EPI3010416	EPI3010422	EPI3010418	EPI3010417	2023-07-04
EPI ISL 17267156	A/European herring gull/Netherlands/8/2023	HPAI	H5N1	2.3.4.4b	BB	EPI2475220	EPI2475221	EPI2475219	EPI2475223	EPI2475216	EPI2475222	EPI2475218	EPI2475217	2023-03-03
EPI ISL 18112333	A/kittiwake/England/330253/2023	HPAI	H5N1	2.3.4.4b	BB	EPI2691234	EPI2691235	EPI2691233	EPI2691237	EPI2691230	EPI2691236	EPI2691232	EPI2691231	2023-07-08
EPI ISL 18112336	A/kittiwake/Scotland/091757/2023	HPAI	H5N1	2.3.4.4b	BB	EPI2691258	EPI2691259	EPI2691257	EPI2691261	EPI2691254	EPI2691260	EPI2691256	EPI2691255	2023-06-30
EPI ISL 18075732	A/black-headed gull/Spain/1509-1-2023 23VIR6502-8/2023	HPAI	H5N1	2.3.4.4b	BB	EPI2671555	EPI2671556	EPI2671554	EPI2671558	EPI2671551	EPI2671557	EPI2671553	EPI2671552	2023-03-16
EPI ISL 18058689	A/black-headed gull/Austria/23063745-001/2023	HPAI	H5N1	2.3.4.4b	BB	EPI2664923	EPI2664924	EPI2664922	EPI2664926	EPI2664919	EPI2664925	EPI2664921	EPI2664920	2023-05-12
EPI ISL 18033241	A/black-headed gull/Poland/MB237-T3/2023	HPAI	H5N1	2.3.4.4b	BB	EPI2652213	EPI2652214	EPI2652212	EPI2648247	EPI2652209	EPI2652215	EPI2652211	EPI2652210	2023-05-30
EPI ISL 17716812	A/black-headed gull/Czech Republic/6577-3/2023	HPAI	H5N1	2.3.4.4b	BB	EPI2577985	EPI2577986	EPI2577984	EPI2577988	EPI2577981	EPI2577987	EPI2577983	EPI2577982	2023-04-24
EPI ISL 7622539	A/Chroicocephalus ridibundus/Belgium/13464/2020	LPAI	H13N8	2.3.4.4b	BB	Excluded*	Excluded*	EPI1942889	Excluded*	EPI1942891	Excluded*	Excluded*	EPI1942894	2021-12-09
EPI ISL 13519451	A/gull/France/22P015977/2022	HPAI	H5N1	2.3.4.4b	BB	EPI2078351	EPI2078352	EPI2078353	EPI2078354	EPI2078355	EPI2078356	EPI2078357	EPI2078358	2022-05-11
EPI ISL 5463797	A/duck/Saratov/29-02V/2021	HPAI	H5N1	2.3.4.4b	AB	EPI1922922	EPI1922923	Excluded*	EPI1922925	Excluded*	EPI1922924	EPI1922920	Excluded*	2021-09-30
EPI ISL 18455202	A/Northern gannet/Norway/2022-07-1146-1T/2022	HPAI	H5N1	2.3.4.4b	C	Excluded*	EPI2782075	Excluded*	EPI2782077	Excluded*	EPI2782079	EPI2782080	Excluded*	2022-06-16
EPI ISL 18851332	A/Northern goshawk/Norway/2023-07-184-1/2023	HPAI	H5N5	2.3.4.4b	I	Excluded*	Excluded*	Excluded*	EPI2972840	Excluded*	Excluded**	EPI2972843	Excluded*	2023-03-13
EPI ISL 17971991	A/domestic cat/Poland/H257-G/2023(H5N1)	HPAI	H5N1	2.3.4.4b	CH	EPI2616171	EPI2616172	Excluded*	EPI2616174	Excluded*	EPI2616173	EPI2616169	Excluded*	2023-06-24
EPI ISL 18292952	A/mink/Finland/506/2023	HPAI	H5N1	2.3.4.4b	BB	EPI2755418	EPI2755417	EPI2755416	EPI2755411	EPI2755414	EPI2755413	EPI2755412	EPI2755415	2023-07-03
EPI ISL 16507095	A/mink/Spain/22VIR12774-13 3869-2/2022	HPAI	H5N1	2.3.4.4b	BB	EPI2291507	EPI2291508	EPI2291506	EPI2291510	EPI2291503	EPI2291509	EPI2291505	EPI2291504	2022-10-26
EPI ISL 17787071	A/black-headed gull/Sweden/SVA230512SZ0306/FB001617/AB-[illegible]	HPAI	H5N1	2.3.4.4b	BB	EPI2589386	EPI2589387	EPI2589388	EPI2589389	EPI2589390	EPI2589391	EPI2589392	EPI2589393	2023-05-11
EPI ISL 18509067	A/herring gull/Latvia/34644 23VIR8835-14/2023	HPAI	H5N1	2.3.4.4b	BB	EPI2794089	EPI2794090	EPI2794088	EPI2794092	EPI2794085	EPI2794091	EPI2794087	EPI2794086	2023-05-29
EPI ISL 18455264	A/Black legged kittiwake/Norway/2023-07-650-2T/2023	HPAI	H5N1	2.3.4.4b	BB	EPI2782185	EPI2782186	EPI2782187	EPI2782188	EPI2782189	EPI2782190	EPI2782191	EPI2782192	2023-05-23
EPI ISL 18939118	A/Herring gull/Norway/2023-07-240-1T/2023	HPAI	H5N1	2.3.4.4b	BB	EPI3077700	EPI3077701	EPI3077699	EPI3077703	EPI3077696	EPI3077702	EPI3077698	EPI3077697	2023-04-12
EPI ISL 18939125	A/Herring gull/Norway/2023-07-263-1k/2023	HPAI	H5N1	2.3.4.4b	BB	missing	missing	missing	EPI3077748	EPI3077744	EPI3077746	EPI3077746	EPI3077745	2023-04-18
EPI ISL 18939119	A/Black-legged kittiwake/Norway/2023-07-651/2023	HPAI	H5N1	2.3.4.4b	BB	EPI3077708	EPI3077709	EPI3077707	EPI3077711	EPI3077704	EPI3077710	EPI3077706	EPI3077705	2023-05-16
EPI ISL 18939120	A/Black-legged kittiwake/Norway/2023-07-793-kt/2023	HPAI	H5N1	2.3.4.4b	BB	EPI3077716	EPI3077717	EPI3077715	EPI3077719	EPI3077712	EPI3077718	EPI3077714	EPI3077713	2023-05-25
EPI ISL 18939126	A/Herring gull/Norway/2023-07-794-1t/2023	HPAI	H5N1	2.3.4.4b	BB	EPI3077752	Excluded*	missing	EPI3077755	EPI3077749	EPI3077754	EPI3077751	EPI3077750	2023-05-26
EPI ISL 18939121	A/Black-legged kittiwake/Norway/2023-07-887-1tr/2023	HPAI	H5N1	2.3.4.4b	BB	EPI3077724	EPI3077725	Excluded*	EPI3077727	EPI3077720	EPI3077726	EPI3077722	EPI3077721	2023-05-25
EPI ISL 18939122	A/Black-legged kittiwake/Norway/2023-07-888-1k/2023	HPAI	H5N1	2.3.4.4b	BB	EPI3077732	EPI3077733	Excluded*	EPI3077735	EPI3077728	EPI3077734	EPI3077730	EPI3077729	2023-06-02
EPI ISL 18939106	A/Black-legged kittiwake/Norway/2023-07-1028-1-2-t/2023	HPAI	H5N1	2.3.4.4b	BB	EPI3077604	EPI3077605	EPI3077603	EPI3077607	EPI3077600	EPI3077606	EPI3077602	EPI3077601	2023-06-07
EPI ISL 18939107	A/Herring gull/Norway/2023-07-1034-1-1k/2023	HPAI	H5N1	2.3.4.4b	BB	EPI3077612	Excluded*	Excluded*	EPI3077615	EPI3077608	EPI3077614	EPI3077610	EPI3077609	2023-06-15
EPI ISL 18939108	A/Black-legged kittiwake/Norway/2023-07-1144-tr/2023	HPAI	H5N1	2.3.4.4b	BB	EPI3077620	EPI3077621	EPI3077619	EPI3077623	EPI3077616	EPI3077622	EPI3077618	EPI3077617	2023-06-15
EPI ISL 18939109	A/Herring gull/Norway/2023-07-1237-t/2023	HPAI	H5N1	2.3.4.4b	BB	EPI3077628	EPI3077629	EPI3077627	EPI3077631	EPI3077624	EPI3077630	EPI3077626	EPI3077625	2023-06-22
EPI ISL 18939110	A/Black-legged kittiwake/Norway/2023-07-1332-1-s/2023	HPAI	H5N1	2.3.4.4b	BB	EPI3077636	EPI3077637	EPI3077635	EPI3077639	EPI3077632	EPI3077638	EPI3077634	EPI3077633	2023-07-13
EPI ISL 18939124	A/Herring gull/Norway/2023-07-1333-1/2023	HPAI	H5N1	2.3.4.4b	BB	EPI3077740	Excluded*	missing	EPI3077743	EPI3077737	EPI3077742	EPI3077739	EPI3077738	2023-06-28
EPI ISL 18939111	A/Black-legged kittiwake/Norway/2023-07-1336-2-s/2023	HPAI	H5N1	2.3.4.4b	BB	EPI3077644	EPI3077645	EPI3077643	EPI3077647	EPI3077640	EPI3077646	EPI3077642	EPI3077641	2023-07-04
EPI ISL 18939112	A/Herring gull/Norway/2023-07-1453-tr/2023	HPAI	H5N1	2.3.4.4b	BB	Excluded*	EPI3077653	EPI3077651	EPI3077655	EPI3077648	EPI3077654	EPI3077650	EPI3077649	2023-07-05
EPI ISL 18939113	A/Black-legged kittiwake/Norway/2023-07-1533-2/2023	HPAI	H5N1	2.3.4.4b	BB	EPI3077660	EPI3077661	EPI3077659	EPI3077663	EPI3077656	EPI3077662	EPI3077658	EPI3077657	2023-07-19
EPI ISL 18939114	A/Black-legged kittiwake/Norway/2023-07-1557-1kl/2023	HPAI	H5N1	2.3.4.4b	BB	EPI3077668	EPI3077669	Excluded*	EPI3077671	EPI3077664	EPI3077670	EPI3077666	EPI3077665	2023-07-20
EPI ISL 18939115	A/Black-legged kittiwake/Norway/2023-07-1640-1tr/2023	HPAI	H5N1	2.3.4.4b	BB	EPI3077676	EPI3077677	EPI3077675	EPI3077679	EPI3077672	EPI3077678	EPI3077674	EPI3077673	2023-07-20
EPI ISL 18939116	A/Black-legged kittiwake/Norway/2023-07-1641-1tr/2023	HPAI	H5N1	2.3.4.4b	BB	EPI3077684	EPI3077685	EPI3077683	EPI3077687	EPI3077680	EPI3077686	EPI3077682	EPI3077681	2023-07-20
EPI ISL 18939117	A/Black-legged kittiwake/Norway/2023-07-1645-1k/2023	HPAI	H5N1	2.3.4.4b	BB	EPI3077692	EPI3077693	EPI3077691	EPI3077695	EPI3077688	EPI3077694	EPI3077690	EPI3077689	2023-07-20
EPI ISL 18939097	A/Black-legged kittiwake/Norway/2023-04-21371-1/2023	HPAI	H5N1	2.3.4.4b	BB	EPI3077532	EPI3077533	EPI3077531	EPI3077535	EPI3077528	EPI3077534	EPI3077530	EPI3077529	2023-07-26
EPI ISL 18939098	A/Black-legged kittiwake/Norway/2023-04-21371-2/2023	HPAI	H5N1	2.3.4.4b	BB	EPI3077540	EPI3077541	EPI3077539	EPI3077543	EPI3077536	EPI3077542	EPI3077538	EPI3077537	2023-07-26
EPI ISL 18939099	A/Black-legged kittiwake/Norway/2023-04-21371-3/2023	HPAI	H5N1	2.3.4.4b	BB	EPI3077548	EPI3077549	EPI3077547	EPI3077551	EPI3077544	EPI3077550	EPI3077546	EPI3077545	2023-07-26
EPI ISL 18939100	A/Black-legged kittiwake/Norway/2023-04-21371-4/2023	HPAI	H5N1	2.3.4.4b	BB	EPI3077556	EPI3077557	EPI3077555	EPI3077559	EPI3077552	EPI3077558	EPI3077554	EPI3077553	2023-07-26
EPI ISL 18939101	A/Black-legged kittiwake/Norway/2023-04-21371-5/2023	HPAI	H5N1	2.3.4.4b	BB	EPI3077564	EPI3077565	EPI3077563	EPI3077567	EPI3077560	EPI3077566	EPI3077562	EPI3077561	2023-07-26
EPI ISL 18939102	A/Black-legged kittiwake/Norway/2023-04-21371-6/2023	HPAI	H5N1	2.3.4.4b	BB	EPI3077572	EPI3077573	EPI3077571	EPI3077575	EPI3077568	EPI3077574	EPI3077570	EPI3077569	2023-07-26
EPI ISL 18939103	A/Black-legged kittiwake/Norway/2023-04-21371-7/2023	HPAI	H5N1	2.3.4.4b	BB	EPI3077580	EPI3077581	EPI3077579	EPI3077583	EPI3077576	EPI3077582	EPI3077578	EPI3077577	2023-07-26
EPI ISL 18939104	A/Black-legged kittiwake/Norway/2023-04-21371-8/2023	HPAI	H5N1	2.3.4.4b	BB	EPI3077588	EPI3077589	EPI3077587	EPI3077591	EPI3077584	EPI3077590	EPI3077586	EPI3077585	2023-07-26
EPI ISL 18939105	A/Black-legged kittiwake/Norway/2023-04-21371-9/2023	HPAI	H5N1	2.3.4.4b	BB	EPI3077596	EPI3077597	EPI3077595	EPI3077599	EPI3077592	EPI3077598	EPI3077594	EPI3077593	2023-07-26
EPI ISL 18939096	A/Black-legged kittiwake/Norway/2023-04-21371-10/2023	HPAI	H5N1	2.3.4.4b	BB	EPI3077524	EPI3077525	EPI3077523	EPI3077527	EPI3077520	EPI3077526	EPI3077522	EPI3077521	2023-07-26

*Sequence excluded due to low or too large distance
**N5 sequence excluded

Collection month	Collection year	Continent/region	Country	County (NO only)	Muncicipality (NO only)	Species (English)	Species (latin)	Family	Order	Originating lab
July	2023	Nordic	Finland			Black-legged kittiwake	*Rissa tridactyla*	*Laridae*	Charadriiformes	Finnish Food Authority
July	2023	Nordic	Finland			Black-legged kittiwake	*Rissa tridactyla*	*Laridae*	Charadriiformes	Finnish Food Authority
July	2023	Nordic	Finland			Black-headed gull	*Chroicocephalus ridibundus*	*Laridae*	Charadriiformes	Finnish Food Authority
July	2023	Nordic	Denmark			Black-headed gull	*Chroicocephalus ridibundus*	*Laridae*	Charadriiformes	Statens Serum Institute
June	2022	Other European	Netherlands			Sandwich tern	*Thalasseus sandvicensis*	*Laridae*	Charadriiformes	Erasmus Medical Center
July	2023	Other European	Netherlands			Sandwich tern	*Thalasseus sandvicensis*	*Laridae*	Charadriiformes	Erasmus Medical Center
March	2023	Other European	Netherlands			Herring gull	*Larus argentatus*	*Laridae*	Charadriiformes	Erasmus Medical Center
July	2023	Other European	England			Black-legged kittiwake	*Rissa tridactyla*	*Laridae*	Charadriiformes	Animal and Plant Health Agency (APHA)
June	2023	Other European	Scotland			Black-legged kittiwake	*Rissa tridactyla*	*Laridae*	Charadriiformes	Animal and Plant Health Agency (APHA)
March	2023	Other European	Spania			Black-headed gull	*Chroicocephalus ridibundus*	*Laridae*	Charadriiformes	Laboratorio Central de Veterinaria
May	2023	Other European	Austria			Black-headed gull	*Chroicocephalus ridibundus*	*Laridae*	Charadriiformes	Austrian Agency for Health and Food Safety (
May	2023	Other European	Poland			Black-headed gull	*Chroicocephalus ridibundus*	*Laridae*	Charadriiformes	National Veterinary Research Institut Poland, I
April	2023	Other European	Czech Republic			Black-headed gull	*Chroicocephalus ridibundus*	*Laridae*	Charadriiformes	State Veterinary Institute Prague
December	2021	Other European	Belgium			Black-headed gull	*Chroicocephalus ridibundus*	*Laridae*	Charadriiformes	Sciensano - Animal Infectious Diseases
May	2022	Other European	France			Gull	*Larus sp.*	*Laridae*	Charadriiformes	Anses (Ploufragan-Plouzané)
September	2021	Other European	Saratov			Domestic duck	*Anas platyrhynchos f. domestica*	*Anatidae*	Anseriformes	Center of Hygiene and Epidemiology in Saratc
June	2022	Nordic	Norway	Hordaland	Bergen	Northern gannet	*Morus bassanus*	*Sulidae*	Suliformes	Norwegian Veterinary Institute
March	2023	Nordic	Norway	Rogaland	Stavanger	Northern goshawk	*Accipiter gentilis*	*Accipitridae*	Accipitriformes	Norwegian Veterinary Institute
June	2023	Other European	Poland			Domestic cat	*Felis catus*	*Felidae*	Carnivora	National Veterinary Research Institut Poland, I
July	2023	Nordic	Finland			American mink	*Neovison vison*	*Mustelidae*	Carnivora	Finnish Food Authority
October	2022	Other European	Spain			American mink	*Neovison vison*	*Mustelidae*	Carnivora	Istituto Zooprofilattico Sperimentale delle Ven
May	2023	Nordic	Sweden			Black-headed gull	*Chroicocephalus ridibundus*	*Laridae*	Charadriiformes	Swedish Veterinary Agency (SVA)
May	2023	Other European	Lativa			Herring gull	*Larus argentatus*	*Laridae*	Charadriiformes	Institute of Food Safety, Animal Health and En
May	2023	Nordic	Norway	Troms	Harstad	Black-legged kittiwake	*Rissa tridactyla*	*Laridae*	Charadriiformes	Norwegian Veterinary Institute
April	2023	Nordic	Norway	Vestland	Bergen	Herring gull	*Larus argentatus*	*Laridae*	Charadriiformes	Norwegian Veterinary Institute
April	2023	Nordic	Norway	Vestland	Bjørnafjorden	Herring gull	*Larus argentatus*	*Laridae*	Charadriiformes	Norwegian Veterinary Institute
May	2023	Nordic	Norway	Troms	Salangen	Black-legged kittiwake	*Rissa tridactyla*	*Laridae*	Charadriiformes	Norwegian Veterinary Institute
May	2023	Nordic	Norway	Troms	Senja	Black-legged kittiwake	*Rissa tridactyla*	*Laridae*	Charadriiformes	Norwegian Veterinary Institute
May	2023	Nordic	Norway	Nordland	Moskenes	Herring gull	*Larus argentatus*	*Laridae*	Charadriiformes	Norwegian Veterinary Institute
May	2023	Nordic	Norway	Troms	Kvæfjord	Black-legged kittiwake	*Rissa tridactyla*	*Laridae*	Charadriiformes	Norwegian Veterinary Institute
June	2023	Nordic	Norway	Finnmark	Hammerfest	Black-legged kittiwake	*Rissa tridactyla*	*Laridae*	Charadriiformes	Norwegian Veterinary Institute
June	2023	Nordic	Norway	Finnmark	Berlevåg	Black-legged kittiwake	*Rissa tridactyla*	*Laridae*	Charadriiformes	Norwegian Veterinary Institute
June	2023	Nordic	Norway	Troms	Tromsø	Herring gull	*Larus argentatus*	*Laridae*	Charadriiformes	Norwegian Veterinary Institute
June	2023	Nordic	Norway	Finnmark	Båtsfjord	Black-legged kittiwake	*Rissa tridactyla*	*Laridae*	Charadriiformes	Norwegian Veterinary Institute
June	2023	Nordic	Norway	Finnmark	Vardø	Herring gull	*Larus argentatus*	*Laridae*	Charadriiformes	Norwegian Veterinary Institute
July	2023	Nordic	Norway	Finnmark	Båtsfjord	Black-legged kittiwake	*Rissa tridactyla*	*Laridae*	Charadriiformes	Norwegian Veterinary Institute
June	2023	Nordic	Norway	Rogaland	Karmøy	Herring gull	*Larus argentatus*	*Laridae*	Charadriiformes	Norwegian Veterinary Institute
July	2023	Nordic	Norway	Finnmark	Alta	Black-legged kittiwake	*Rissa tridactyla*	*Laridae*	Charadriiformes	Norwegian Veterinary Institute
July	2023	Nordic	Norway	Nordland	Bodø	Herring gull	*Larus argentatus*	*Laridae*	Charadriiformes	Norwegian Veterinary Institute
July	2023	Nordic	Norway	Nord-Trøndelag	Nærøysund	Black-legged kittiwake	*Rissa tridactyla*	*Laridae*	Charadriiformes	Norwegian Veterinary Institute
July	2023	Nordic	Norway	Finnmark	Vadsø	Black-legged kittiwake	*Rissa tridactyla*	*Laridae*	Charadriiformes	Norwegian Veterinary Institute
July	2023	Nordic	Norway	Finnmark	Vadsø	Black-legged kittiwake	*Rissa tridactyla*	*Laridae*	Charadriiformes	Norwegian Veterinary Institute
July	2023	Nordic	Norway	Finnmark	Vardø	Black-legged kittiwake	*Rissa tridactyla*	*Laridae*	Charadriiformes	Norwegian Veterinary Institute
July	2023	Nordic	Norway	Rogaland	Karmøy	Black-legged kittiwake	*Rissa tridactyla*	*Laridae*	Charadriiformes	Norwegian Veterinary Institute
July	2023	Nordic	Norway	Finnmark	Vadsø	Black-legged kittiwake	*Rissa tridactyla*	*Laridae*	Charadriiformes	Norwegian Veterinary Institute
July	2023	Nordic	Norway	Finnmark	Vadsø	Black-legged kittiwake	*Rissa tridactyla*	*Laridae*	Charadriiformes	Norwegian Veterinary Institute
July	2023	Nordic	Norway	Finnmark	Vadsø	Black-legged kittiwake	*Rissa tridactyla*	*Laridae*	Charadriiformes	Norwegian Veterinary Institute
July	2023	Nordic	Norway	Finnmark	Vadsø	Black-legged kittiwake	*Rissa tridactyla*	*Laridae*	Charadriiformes	Norwegian Veterinary Institute
July	2023	Nordic	Norway	Finnmark	Vadsø	Black-legged kittiwake	*Rissa tridactyla*	*Laridae*	Charadriiformes	Norwegian Veterinary Institute
July	2023	Nordic	Norway	Finnmark	Vadsø	Black-legged kittiwake	*Rissa tridactyla*	*Laridae*	Charadriiformes	Norwegian Veterinary Institute
July	2023	Nordic	Norway	Finnmark	Vadsø	Black-legged kittiwake	*Rissa tridactyla*	*Laridae*	Charadriiformes	Norwegian Veterinary Institute
July	2023	Nordic	Norway	Finnmark	Vadsø	Black-legged kittiwake	*Rissa tridactyla*	*Laridae*	Charadriiformes	Norwegian Veterinary Institute
July	2023	Nordic	Norway	Finnmark	Vadsø	Black-legged kittiwake	*Rissa tridactyla*	*Laridae*	Charadriiformes	Norwegian Veterinary Institute
July	2023	Nordic	Norway	Finnmark	Vadsø	Black-legged kittiwake	*Rissa tridactyla*	*Laridae*	Charadriiformes	Norwegian Veterinary Institute

Submitting lab	Authors
Finnish Food Authority	Kauppinen, Ari; Kareinen, Lauri; Tammiranta, Niina; Kantala, Tuija; Fusaro, Alice; Giussani, Edoardo; Gadd, Tuija
Finnish Food Authority	Kauppinen, Ari; Kareinen, Lauri; Tammiranta, Niina; Kantala, Tuija; Fusaro, Alice; Giussani, Edoardo; Gadd, Tuija
Finnish Food Authority	Kauppinen, Ari; Kareinen, Lauri; Tammiranta, Niina; Kantala, Tuija; Fusaro, Alice; Galiano, Monica; Ikonen, Niina; Lindh, Erika; Gadd, Tuija
Statens Serum Institute	Hjulsager,Charlotte; Liang,Yuan
Erasmus Medical Center	Not stated
Erasmus Medical Center	Bellido, Beatriz
Erasmus Medical Center	Not stated
Animal and Plant Health Agency (APHA)	Not stated
Animal and Plant Health Agency (APHA)	Not stated
Istituto Zooprofilattico Sperimentale Delle Venezie	Ruano,M.J.; Rocha,A.; Sanchez,A.; Aguero,M.; Pastori, A.; Zecchin, B.; Fusaro, A.; Schivo, A.; Salviato, A.; Palumbo, E.; Giussani, E.; Monne, I.; Terregino, C.
Austrian Agency for Health and Food Safety (AGES)	Not stated
National Veterinary Research Institut Poland, PIWet-PIB	Swieton,Edyta; Tarasiuk,Karolina; Wyrostek,Krzysztof; Kozak,Edyta; Stys-Fijol,Natalia; Smietanka,Krzysztof
State Veterinary Institute Prague	Alexander,Nagy; Lenka,Cernikova; Martina,Stara
Sciensano, Department of Animal Infectious Diseases	Van Borm, Steven; Vandenbussche, Frank; Roupie, Virginie; Lambrecht, Benedicte; Van Borm, Steven; Vandenbussche, Frank; Roupie, Virginie; Lambrecht, Benedicte; Steensels, Mieke
ANSES Agence Nationale De Securite Sanitaire De L'alim	Not stated
State Research Center of Virology and Biotechnology (VEC	Natalia,Goncharova; Ivan,Susloparov; Natalia,Kolosova; Alexey,Danilenko; Juliya,Bulanovich; Vasiliy,Marchenko; Alexander,Ryzhikov
Norwegian Veterinary Institute	Gjerset, Britt; Bøe, Cathrine A.
Norwegian Veterinary Institute	Gjerset, Britt; Bøe, Cathrine A.
National Veterinary Research Institut Poland, PIWet-PIB	Swieton, E.; Domanska-Blicharz, K.; Tarasiuk, K.; Wyrostek, K.; Stys-Fijol, N.
Finnish Food Authority	Kauppinen, Ari; Kareinen, Lauri; Tammiranta, Niina; Kantala, Tuija; Fusaro, Alice; Gadd, Tuija
Istituto Zooprofilattico Sperimentale Delle Venezie	Ruano,M.J.; Rocha,A.; Sanchez,A.; Aguero,M.; Pastori, A.; Zecchin, B.; Fusaro, A.; Schivo, A.; Salviato, A.; Palumbo, E.; Giussani, E.; Monne, I.; Terregino, C.
Swedish Veterinary Agency (SVA)	Siamak,Zohari
Istituto Zooprofilattico Sperimentale Delle Venezie	Krivko, L.; Pastori, A.; Zecchin, B.; Fusaro, A.; Schivo, A.; Salviato, A.; Palumbo, E.; Giussani, E.; Monne, I.; Terregino, C.
Norwegian Veterinary Institute	Gjerset, Britt; Bøe, Cathrine A.
Norwegian Veterinary Institute	Gjerset, Britt; Bøe, Cathrine A.
Norwegian Veterinary Institute	Gjerset, Britt; Bøe, Cathrine A.
Norwegian Veterinary Institute	Gjerset, Britt; Bøe, Cathrine A.
Norwegian Veterinary Institute	Gjerset, Britt; Bøe, Cathrine A.
Norwegian Veterinary Institute	Gjerset, Britt; Bøe, Cathrine A.
Norwegian Veterinary Institute	Gjerset, Britt; Bøe, Cathrine A.
Norwegian Veterinary Institute	Gjerset, Britt; Bøe, Cathrine A.
Norwegian Veterinary Institute	Gjerset, Britt; Bøe, Cathrine A.
Norwegian Veterinary Institute	Gjerset, Britt; Bøe, Cathrine A.
Norwegian Veterinary Institute	Gjerset, Britt; Bøe, Cathrine A.
Norwegian Veterinary Institute	Gjerset, Britt; Bøe, Cathrine A.
Norwegian Veterinary Institute	Gjerset, Britt; Bøe, Cathrine A.
Norwegian Veterinary Institute	Gjerset, Britt; Bøe, Cathrine A.
Norwegian Veterinary Institute	Gjerset, Britt; Bøe, Cathrine A.
Norwegian Veterinary Institute	Gjerset, Britt; Bøe, Cathrine A.
Norwegian Veterinary Institute	Gjerset, Britt; Bøe, Cathrine A.
Norwegian Veterinary Institute	Gjerset, Britt; Bøe, Cathrine A.
Norwegian Veterinary Institute	Gjerset, Britt; Bøe, Cathrine A.
Norwegian Veterinary Institute	Gjerset, Britt; Bøe, Cathrine A.
Norwegian Veterinary Institute	Gjerset, Britt; Bøe, Cathrine A.
Norwegian Veterinary Institute	Rødmo, Grim; Gjerset, Britt; Bøe, Cathrine A.
Norwegian Veterinary Institute	Rødmo, Grim; Gjerset, Britt; Bøe, Cathrine A.
Norwegian Veterinary Institute	Rødmo, Grim; Gjerset, Britt; Bøe, Cathrine A.
Norwegian Veterinary Institute	Rødmo, Grim; Gjerset, Britt; Bøe, Cathrine A.
Norwegian Veterinary Institute	Rødmo, Grim; Gjerset, Britt; Bøe, Cathrine A.
Norwegian Veterinary Institute	Rødmo, Grim; Gjerset, Britt; Bøe, Cathrine A.
Norwegian Veterinary Institute	Rødmo, Grim; Gjerset, Britt; Bøe, Cathrine A.
Norwegian Veterinary Institute	Rødmo, Grim; Gjerset, Britt; Bøe, Cathrine A.
Norwegian Veterinary Institute	Rødmo, Grim; Gjerset, Britt; Bøe, Cathrine A.
Norwegian Veterinary Institute	Rødmo, Grim; Gjerset, Britt; Bøe, Cathrine A.
