# Supplementary material for: Highly Pathogenic Avian Influenza A (H5N1) Caused Mass Death Among Black‐Legged Kittiwakes (Rissa tridactyla) in Norway, 2023
Source: Transbound Emerg Dis. 2026 Feb 18;2026:2963364. doi: 10.1155/tbed/2963364 (PMC12917261; doi:10.1155/tbed/2963364)
Supplement: Supplementary file 4 — Supporting Information 4 Appendix 3: Table showing markers which might indicate mammalian adaptation identified using FluMut in whole genome sequenced HPAI H5N1 clade 2.3.4.4b genotype BB viruses from 10 black‐legged kittiwakes at Ekkerøy in Norway, 2023. [file TBED-2026-2963364-s004.pdf]

Appendix 3. Table 1. Markers which might indicate mammalian adaptation identified using FluMut* in whole genome sequenced HPAI H5N1 clade 2.3.4.4b genotype BB viruses from 10 black-legged kittiwakes at Ekkerøy in Norway, 2023.

Protein	Marker	Effect	Subtype	References
PB2	L89V, G309D	Increased polymerase activity in mammalian cells, Increased virulence in mice	H5N1	Li J. et al., 2009; Suttie A. et al., 2019
PB2	K389R	Increased polymerase activity in mammalian cells, Increased replication in mammalian cells	H7N9	Hu M. et al., 2017b; Suttie A. et al., 2019
PB2	L89V, G309D, T339K, R477G, I495V, K627E, A676T	Increased polymerase activity in mammalian cells, Increased virulence in mice	H5N1	Li J. et al., 2009; Suttie A. et al., 2019
PB2	V598T	Increased replication in mammalian cells, Increased polymerase activity in mammalian cells, Increased virulence in mice	H7N9	Hu M. et al., 2017b; Suttie A. et al., 2019
PB2	K699R	Enhanced virulence in mice, Increased viral replication in mammalian cells	H1N1	Zhang T. et al., 2017
PB1	D3V	Increased polymerase activity and replication in avian and mammalian cells	H5N1	Elgendy E. et al., 2017; Suttie A. et al., 2019
PB1	D622G	Increased polymerase activity in mammalian cells, increased polymerase activity and virulence in mice	H5N1	Feng X. et al., 2016; Suttie A. et al., 2019
PB1-F2	N66S	Enhanced antiviral response, replication and virulence in mice	H5N1	Conenello G. et al., 2007; Schmolke M. et al., 2011; Suttie A. et al., 2019
PA	S37A	Increased polymerase activity in mammalian cells	H7N9	Suttie A. et al., 2019; Yamayoshi S. et al., 2014
PA	N383D	Increased polymerase activity in avian and mammalian cells	H5N1	Song J. et al., 2011; Song J. et al., 2015; Suttie A. et al., 2019
PA	N409S	Increased polymerase activity and replication in mammalian cells	H7N9	Suttie A. et al., 2019; Yamayoshi S. et al., 2014
HA1-5	S107R, T108I	Increased pH of fusion, increased virulence in chickens and mice	H5N1	Suttie A. et al., 2019; Wessels U. et al., 2018
HA1-5	S133A	Increased pseudovirus binding to α2-6	H5N1	Suttie A. et al., 2019; Yang Z. et al., 2007
HA1-5	T134A	Increased viral replication in mice lungs, Increased virus thermostability	H9N2	Zhang J. et al., 2023
HA1-5	S154N	Increased virus binding to α2-6	H5N1	Suttie A. et al., 2019; Wang W. et al., 2010
HA1-5	T156A	Increased transmission in guinea pigs, Increased virus binding to α2-6	H5N1	Gao Y. et al., 2009; Suttie A. et al., 2019; Wang W. et al., 2010
HA1-5	V182N	Decreased virus binding to α2-3, Increased virus binding to α2-6	H13N6	Lu X. et al., 2013; Suttie A. et al., 2019
HA1-5	K218Q, S223R	Increased virus binding to α2-3 and α2-6	H5N1	Guo H. et al., 2017; Suttie A. et al., 2019
HA2-5	K64E	Decreased HA stability, Decreased virulence in mice, Increased pH of fusion	H7N9	Sun X. et al., 2019; Suttie A. et al., 2019
NA	A369I	Distruption of the second sialic acid binding site (2SBS)	H5N1, Unknown	de Vries E. et al., 2023, Du W. et al., 2018
NP	Y52N	Evade human BTN3A3 (inhibitor of avian influenza A viruses replication)	Unknown	Pinto R. et al., 2023
M2	A30S	Increased resistance to amantadine and rimantadine	H5N1, H5N2, H7N2	Bean W. et al., 1989; Cheung C. et al., 2006; Ilyushina N. et al., 2005; Suttie A. et al., 2019
NS1	P42S	Decreased antiviral response, and increased virulence in mice	H5N1	Jiao P. et al., 2008; Suttie A. et al., 2019
NS1	L103F, I106M	Increased virulence in mice	H5N1	Kuo R. et al., 2009; Spesock A. et al., 2011; Suttie A. et al., 2019
NS1	I106M	Increased viral replication in mammalian cells, Increased virulence in mice	H1N1 with all internal genes from H7N9	Ayllon J. et al., 2014; Suttie A. et al., 2019
NS1	C138F	Decreased interferon response, Increased viral replication in mammalian cells	H5N1	Li J. et al., 2018; Suttie A. et al., 2019
NS1**	K55E, K66E, C138F	Decreased interferon response, Enhanced replication in mammalian cells	H5N1	Li J. et al., 2018; Suttie A. et al., 2019
NS1, NS2	NS1-205S, NS-2:T48A	Decreased antiviral response in ferrets	H5N1	Imai H. et al., 2010; Suttie A. et al., 2019

*https://github.com/izsvenezie-virology/FluMut, FluMutGUI 3.1.1; FluMut 0.6.3; FluMutDB 6.3, released on 2024-09-12
**present in all viruses, except one (bird no. 9 K55G, K66E, C138F)
